# Supplementary figures and images for: The PAX-FOXO1s trigger fast trans-differentiation of chick embryonic neural cells into alveolar rhabdomyosarcoma with tissue invasive properties limited by S phase entry inhibition
Source: PLoS Genet. 2020 Nov 11;16(11):e1009164. doi: 10.1371/journal.pgen.1009164 (PMC7682867; doi:10.1371/journal.pgen.1009164)

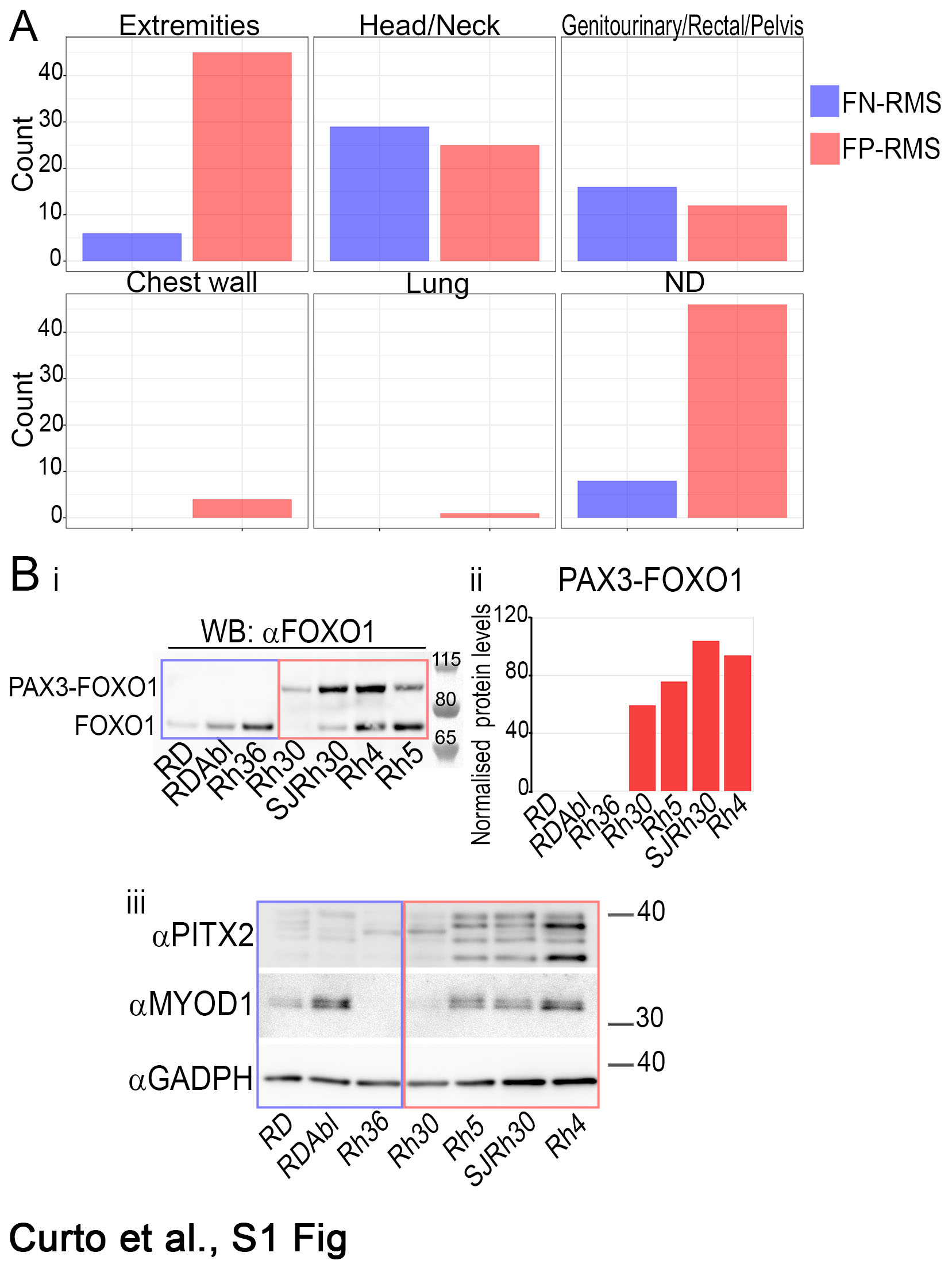

Supplement: S1 Fig — (A) Body locations of RMS biopsies. Locations of FP-RMS (red) and FN-RMS (blue) biopsies whose transcriptome has been assessed in Fig 2A and coming from previous studies [42–46]. ND: Non determined. (B) PITX2 expression distinguishes FP-RMS from FN-RMS cells. (i, iii) Pictures of western blots using the indicated antibodies on proteins extracted from the indicated FN-RMS (RD, RDAbl, Rh36) and FP-RMS (Rh3, Rh5, SJRH30, Rh4) cell lines and (ii) normalized PAX3-FOXO1 levels to that of GAPDH. This shows variable levels of PAX3-FOXO1 (i, ii) between FP-RMS cell lines and of MYOD1 across all RMS cell lines (iii). In addition, specific ectopic expression of several PITX2 isoforms (iii) in FP-RMS versus FN-RMS cell lines is revealed (see also S1 to S4 Raw images). (TIF) [file pgen.1009164.s001.tif]

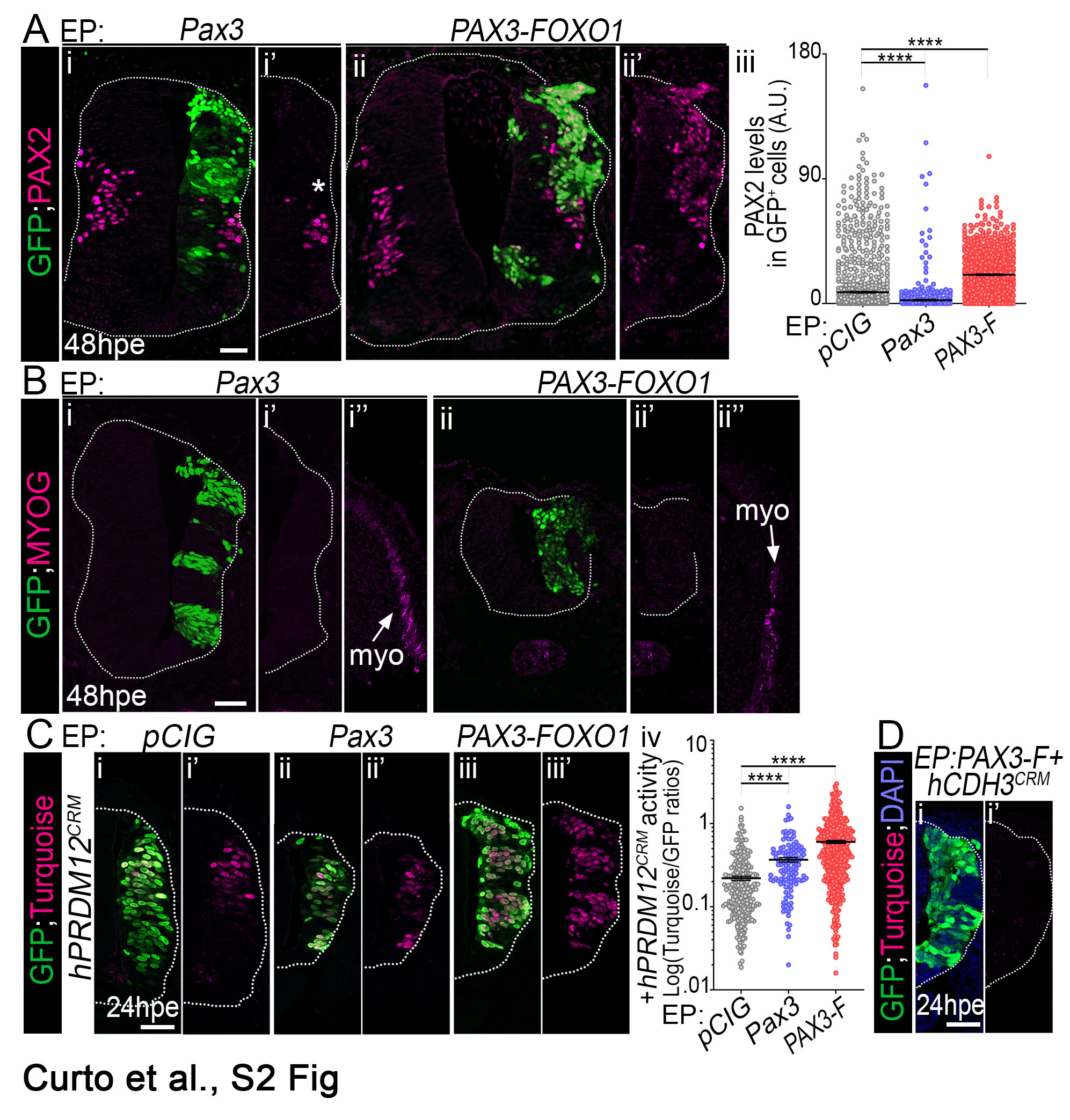

Supplement: S2 Fig — (A) (i-ii') Immunodetection of GFP and PAX2 on transverse sections of chick embryos 48hpe with the indicated plasmids. (iii) Quantification of PAX2 expression levels in GFP+ cells in the spinal cords of chick embryos 48hpe with the indicated constructs (dots: single cell values; bars: mean ± s.e.m.; n>5 embryos). (B) Immunodetection of GFP and MYOG on transverse sections of chick embryos 48hpe with the indicated plasmids. x” panels are views on the myotome (myo) of x panel embryo. (Ci-iii’, D) Immunostaining for GFP, Turquoise direct fluorescence and DAPI staining on transverse sections of chick embryos 24hpe with the indicated plasmids and a reporter for human PRDM12CRM and CDH3CRM. (Civ) Quantification of Turquoise levels normalised to that of GFP in cells electroporated with PRDM12CRM reporter at 24hpe (dots: single cell values; bars: mean ± s.e.m.; n>4 embryos). Mann-Whitney U test p-value: ****: p< 0.0001. Scale bars: 50μm. (TIF) [file pgen.1009164.s002.tif]

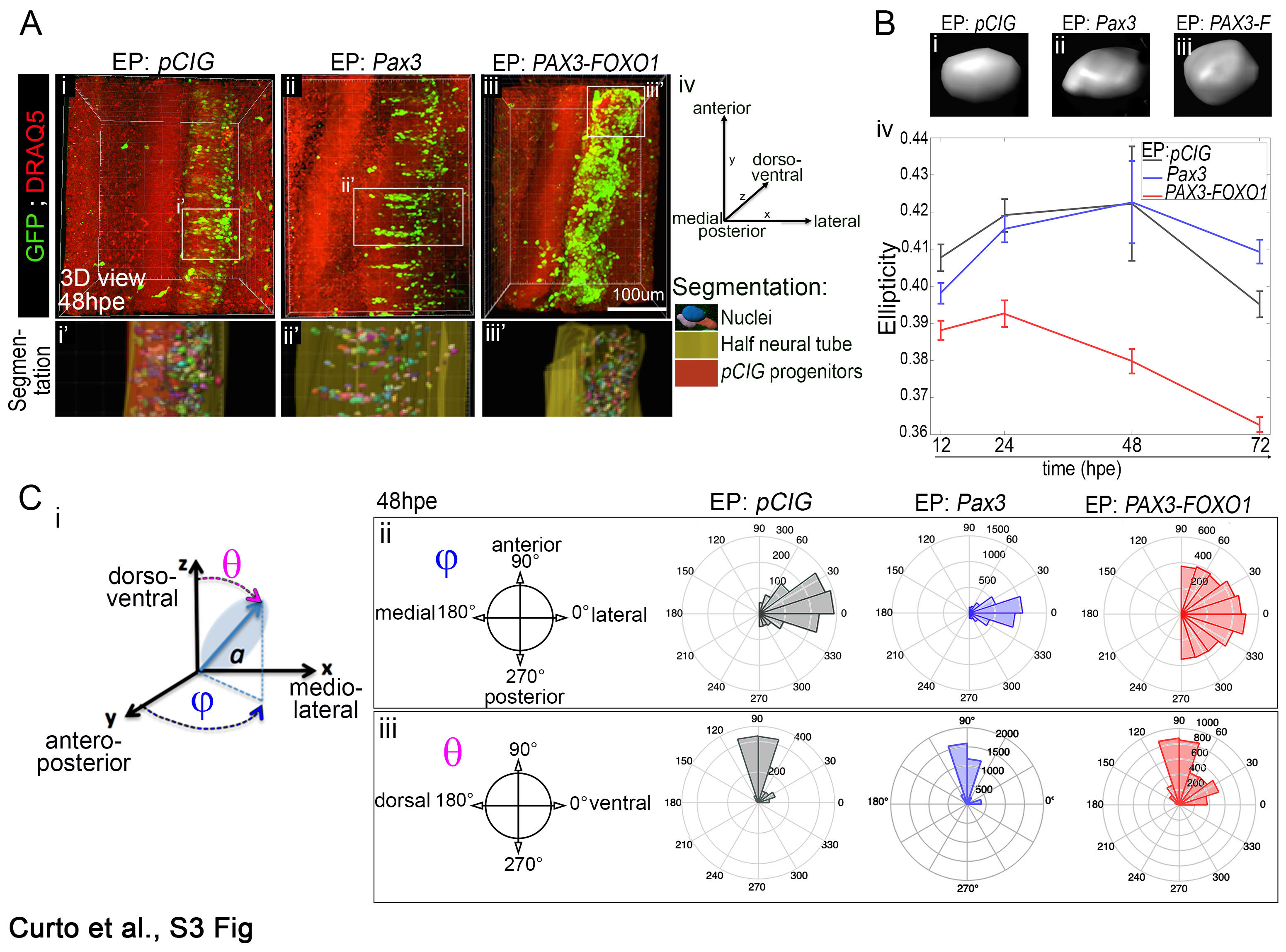

Supplement: S3 Fig — (A) (i-iii) Projection of 3D images of embryos 48hpe with the indicated plasmids, stained with DRAQ5 and immunolabelled for GFP. (i’-iii’) Result of the segmentation performed at the level of the boxes indicated on samples i-iii. Surfaces delimiting the electroporated half of the neural tube are transparent yellow, while cell nuclei are coloured. In pCIG sample, the surface segregating progenitor nuclei from neurons is highlighted in transparent red. (iv) x (medial-lateral), y (antero-posterior) and z (dorsal-ventral) axes giving the orientation of i-iii samples. (B) (i-iii) Representative 3D shape of GFP+ nuclei segmented from scanned whole embryos 48hpe with the indicated plasmids. (iv) Temporal dynamics of the ellipticity of nuclei measured from the segmentation of GFP+ nuclei (as shown in i-iii) in whole-mount embryos (mean ± s.d., n>6 embryos). (C) (i) Representation in the 3 dimensions of the chick embryos of θ and φ polar angles of the vector (blue arrow) defining the major axis of a cell ellipsoid fit (blue circle). (ii-iii) φ (ii) and θ (iii) possible values and major axes of chick embryos (black circles) and measured values in embryos electroporated with the indicated plasmids at 48hpe. (TIF) [file pgen.1009164.s003.tif]

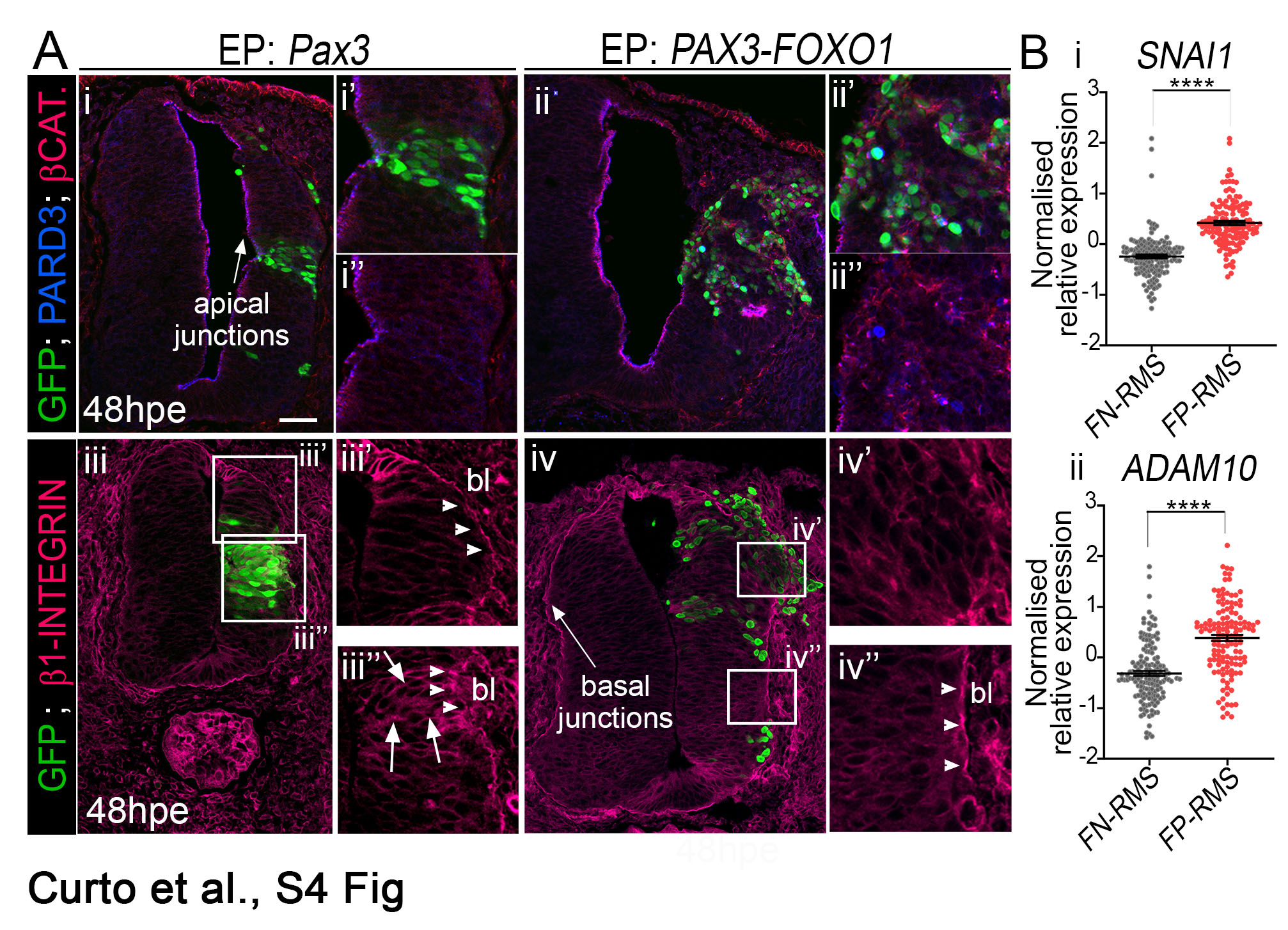

Supplement: S4 Fig — (A) Immunodetection of GFP, PARD3, activated βCATENIN (βCAT.) and β1-INTEGRIN on transverse sections of chick embryos 48hpe with the indicated plasmids. In i and ii, x’ and x” panels are blown up on a subset of x panel GFP+ cells; in iii and iv the x and x” are blown up on a subset of x panel GFP+ and GFP- cells. Arrowheads in x’ panels point are the accumulation of β1-INTEGRIN on the basal side of cells. Arrows in iii’ indicate increased levels of β1-INTEGRIN at the membrane of GFP+ cells. bl: basal lamina. Scale bars: 50μm. (B) Normalized levels of SNAI1 and ADAM10 mRNA assayed by DNA microarrays in FP-RMS and FN-RMS biopsies (dots: RNA sample values; bars: mean ± s.e.m.; Mann-Whitney U test p-value: ****: p<0.0001). (TIF) [file pgen.1009164.s004.tif]

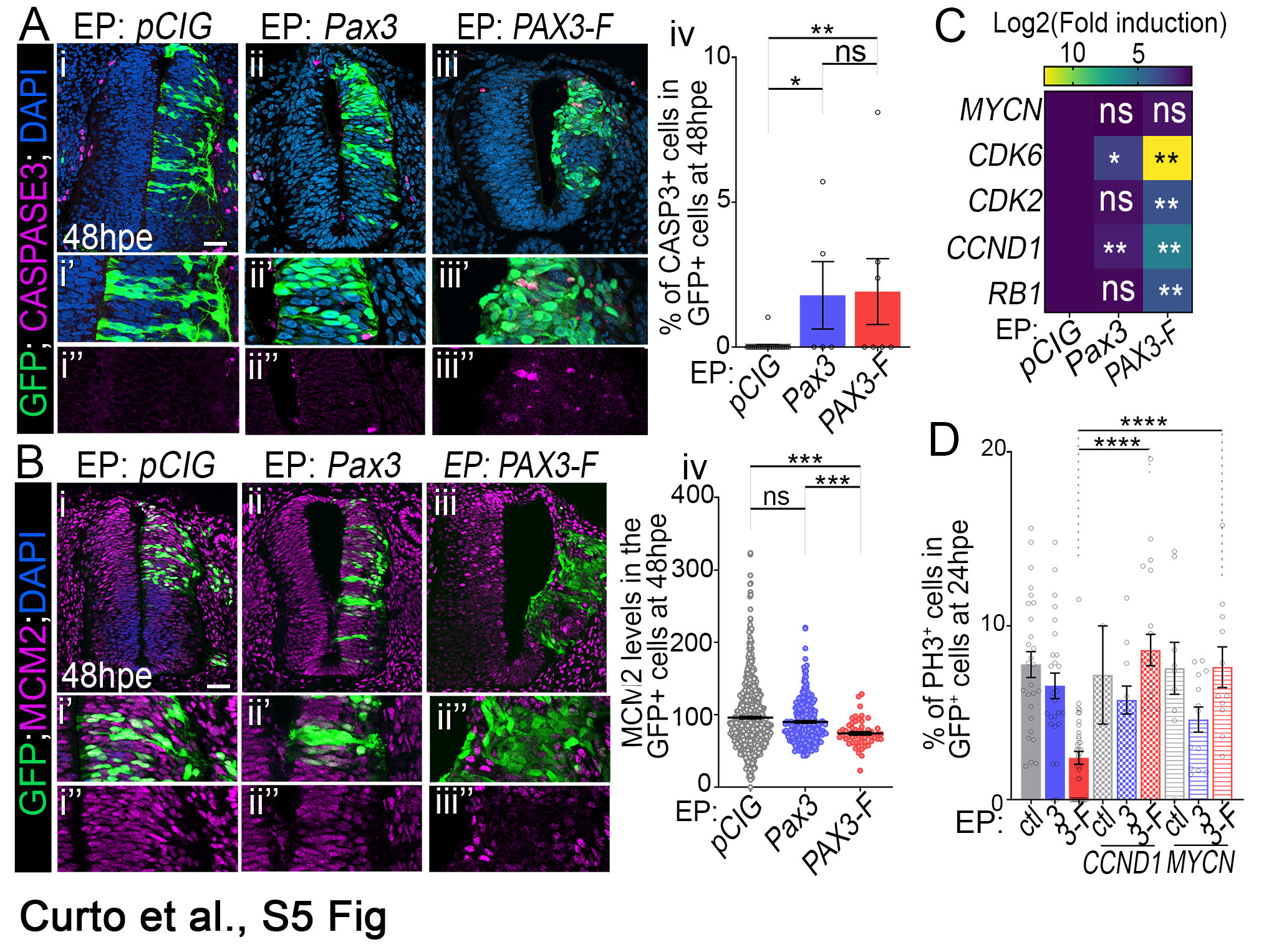

Supplement: S5 Fig — (A) (i-iii”) GFP and activated CASPASE3 immunodetection and DAPI staining on transverse section of chick embryos 48hpe with the indicated plasmids. (iv) Quantification of the number of activated CASPASE3+ cells in the GFP+ population in embryos 48hpe with the indicated plasmids (dots: embryo values; bar plots: mean ± s.e.m.). (B) GFP and MCM2 immunodetection and DAPI staining on transverse sections of chick embryos 48hpe with the indicated plasmids. (iv) Quantification of MCM2 levels in the GFP+ cells 48hpe with the indicated plasmids (dots: single cell values; bar plots: mean ± s.e.m.; n>5 embryos). (C) Heatmaps indicated fold changes in the expression of the indicated genes relative to their mean expression in pCIG samples assayed in FAC sorted GFP+ from chick embryos 48hpe with the indicated constructs. (D) Quantification of the number of PH3+ cells in the GFP+ population in embryos 24hpe with the indicated plasmids (dots: embryo values; bar plots: mean ± s.e.m.). x’ and x” panels are blown up on a subset of x panel GFP+ cells. Mann-Whitney U test p-value: *: p< 0.05, **: p<0.01, ***: p<0.001, ****: p<0.0001, ns: p>0.05. Scale bars: 50μm. (TIF) [file pgen.1009164.s005.tif]

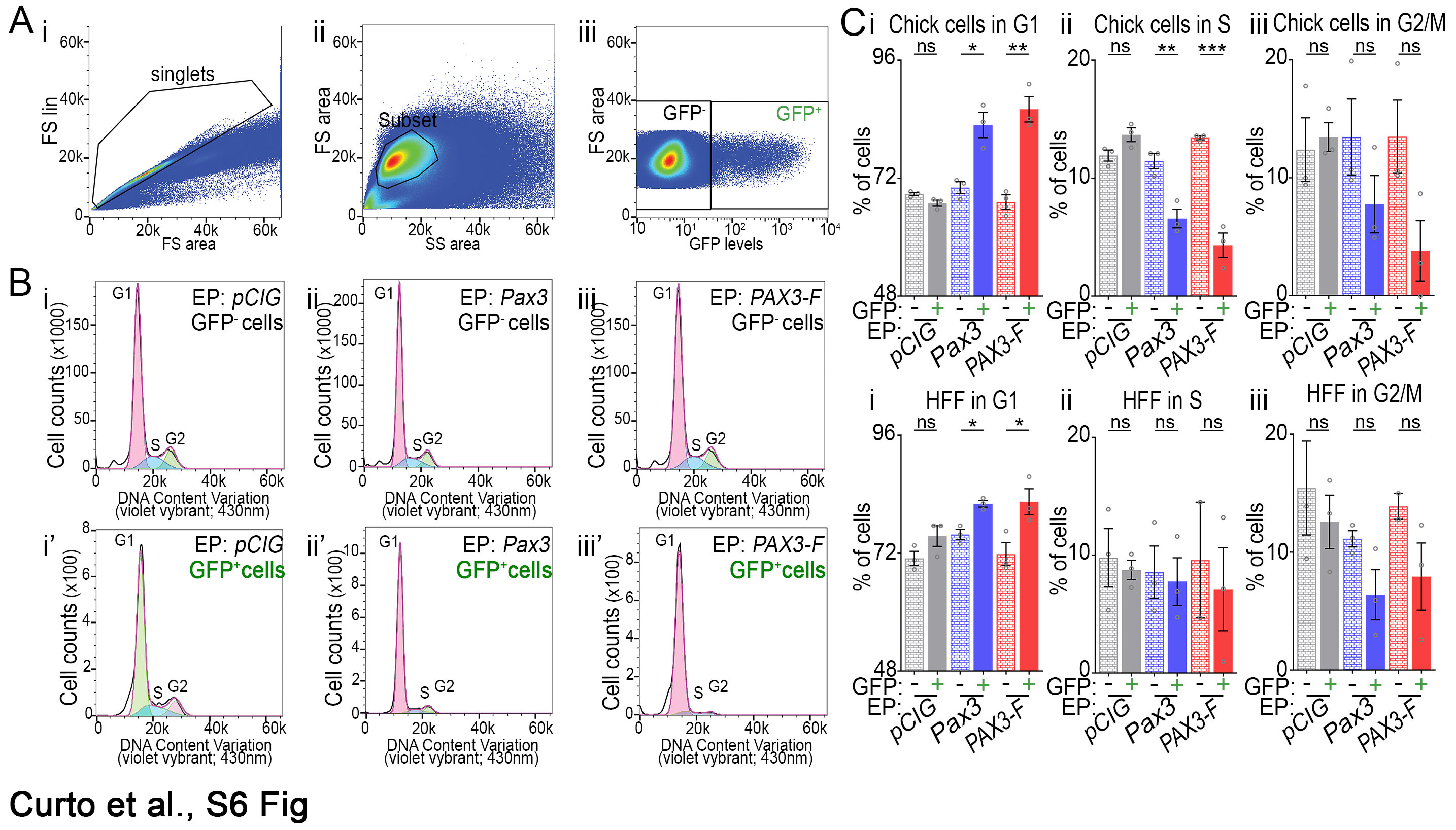

Supplement: S6 Fig — (A) FACS gating strategy in 3 steps using Flowjo: (i) isolation of singlets (FS: forward scatter/approximation of cell size; lin: linear); (ii) selection of cells based on their size (FS: forward scatter) and granularity (SS: side scatter); (iii) segregation of GFP+ from the GFP- pools. (B) FACS plots showing DNA content distribution of GFP- (i,ii,iii) and GFP+ (i',ii’,iii’) chick neural cells stained with Vybrant DyeCycle Violet stain (black line) and the Dean/Jett/Fox model based cell cycle phases gating (pink line: extrapolation of the distribution with the model; pink area: G0/G1 phase, blue area: S phase and green area: G2/M phase) in chick embryos 48hpe with the indicated plasmids. (C) Proportion of cells in the indicated cell cycle phase assayed by DNA content distribution of FAC sorted GFP- and GFP+ chick neural (i-iii) and Human Forskin Fibroblats (HFF; iv-vi) stained with Vybrant DyeCycle Violet stain (dots: mean value on cells analysed on independent FAC sorted samples; bar plots: mean ± s.e.m., unpaired test p-value: *: p< 0.05, **: p<0.01, ***: p<0.001, ns: p>0.05). (TIF) [file pgen.1009164.s006.tif]

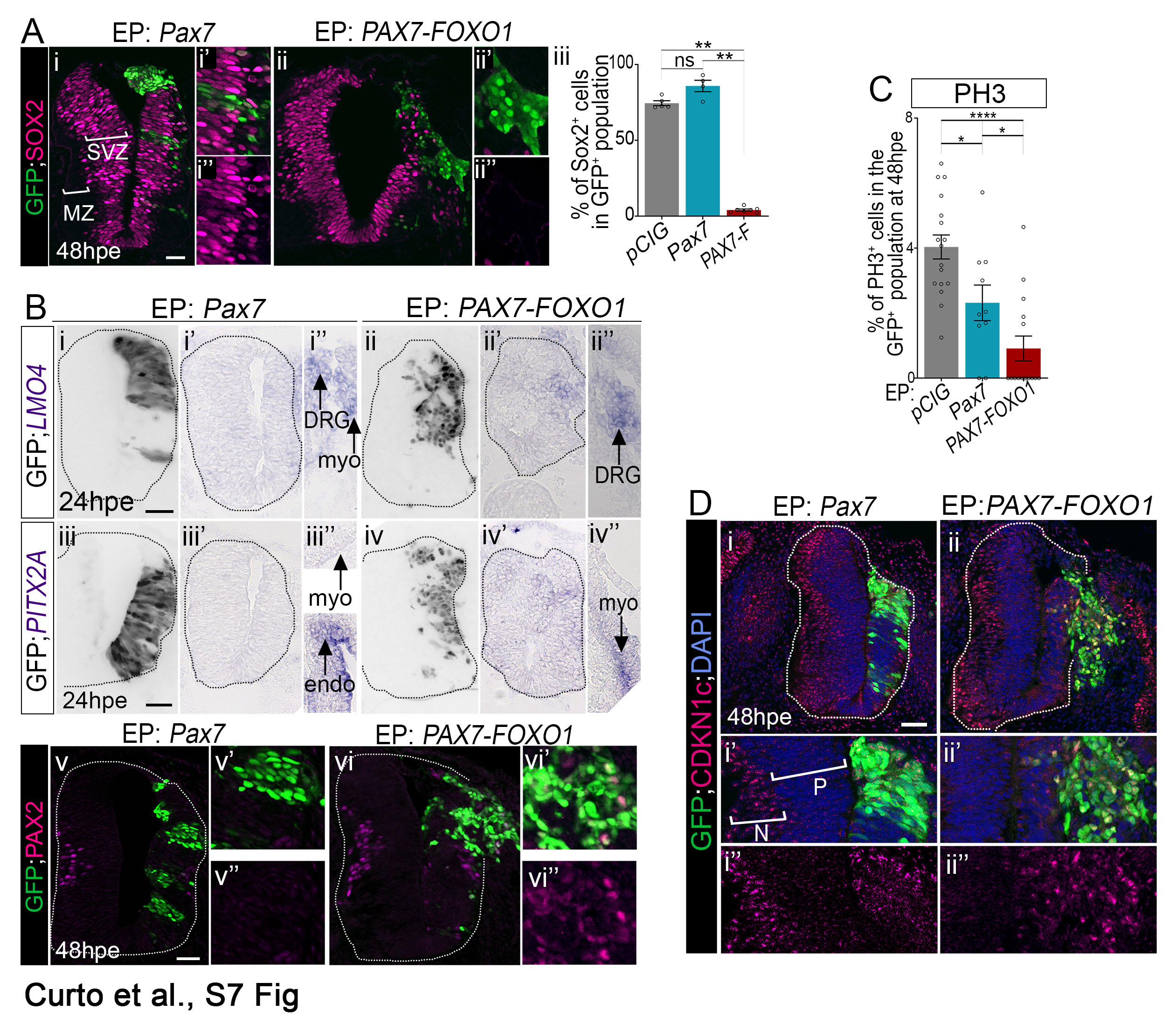

Supplement: S7 Fig — (A) (i-ii”) Immunodetection of GFP and SOX2 on transverse sections of chick embryos 48hpe with the indicated plasmids. MZ: Mantle Zone; SVZ: Sub-Ventricular Zone. (iii) Percentage of SOX2+ cells in the GFP+ population 48hpe with the indicated plasmids (dots: embryo values; bar plots: mean ± s.e.m. (B) In situ hybridization for LMO4 (i-ii”), PITX2a (iii-iv”) and immuno-detection of GFP and PAX2 (v-vi”) on transverse sections of chick embryos 24hpe (i-iv”) or 48hpe (v-vi’) with the indicated plasmids. x” panels in i-iv in display region of the DRG, somite or endoderm regions of x sample. DRG: dorsal root ganglia; endo: endoderm; myo: myotome. (C) Quantification of the number of PH3+ cells in the GFP+ cells in embryos 48hpe with the indicated plasmids. (D) Immunodetection of GFP and CDKN1c on transverse sections of chick embryos 48hpe with the indicated plasmids. P: progenitors; N: neurons. x’ and x” panels are blown up on a subset of x panel GFP+ cells. Mann-Whitney U test p-values: *: p<0.05, **: p< 0.01, ****: p<0.0001, ns: p>0.05. Scale bars: 50μm. (TIF) [file pgen.1009164.s007.tif]

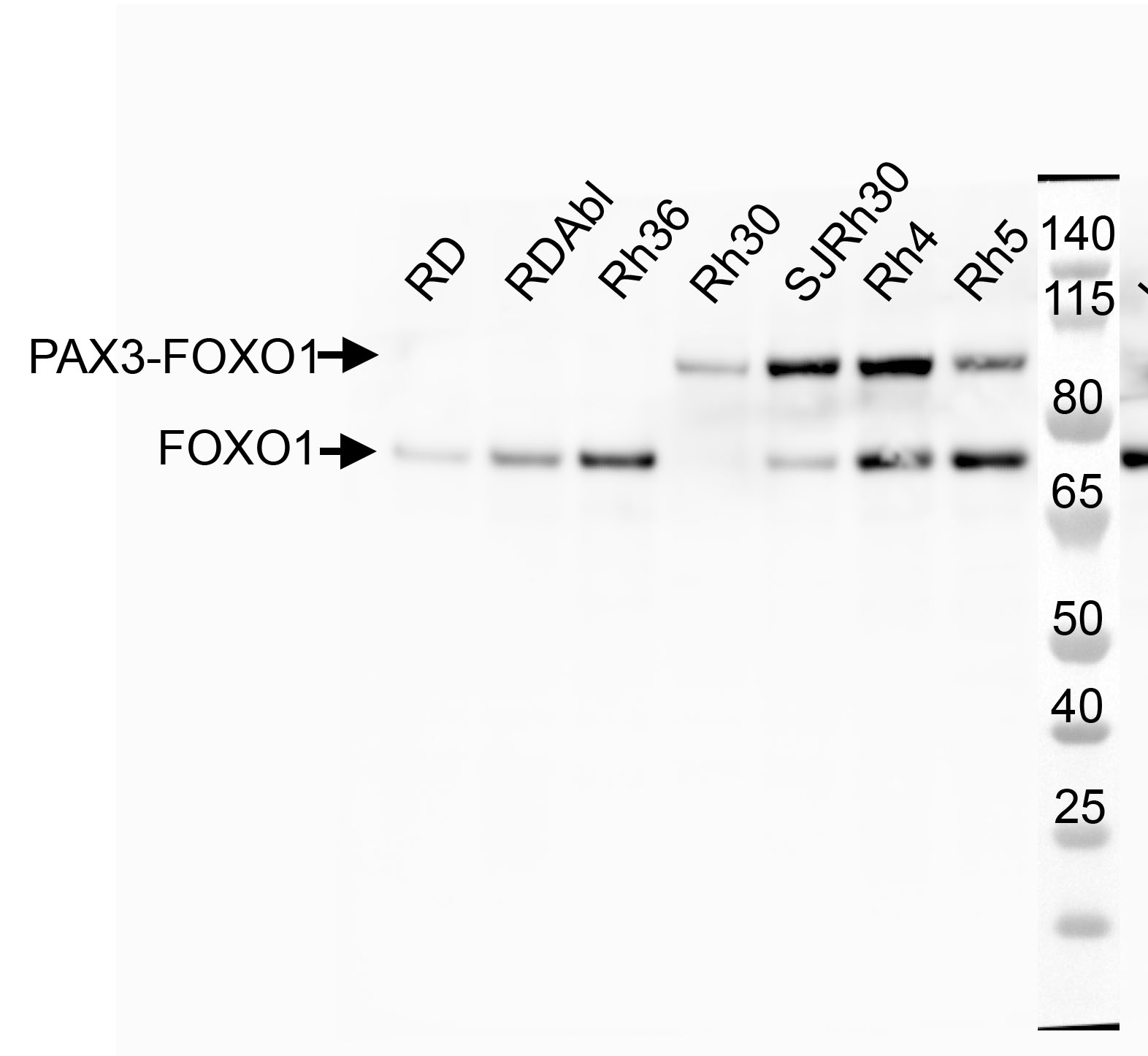

Supplement: S1 Raw image — (TIF) [file pgen.1009164.s015.tif]

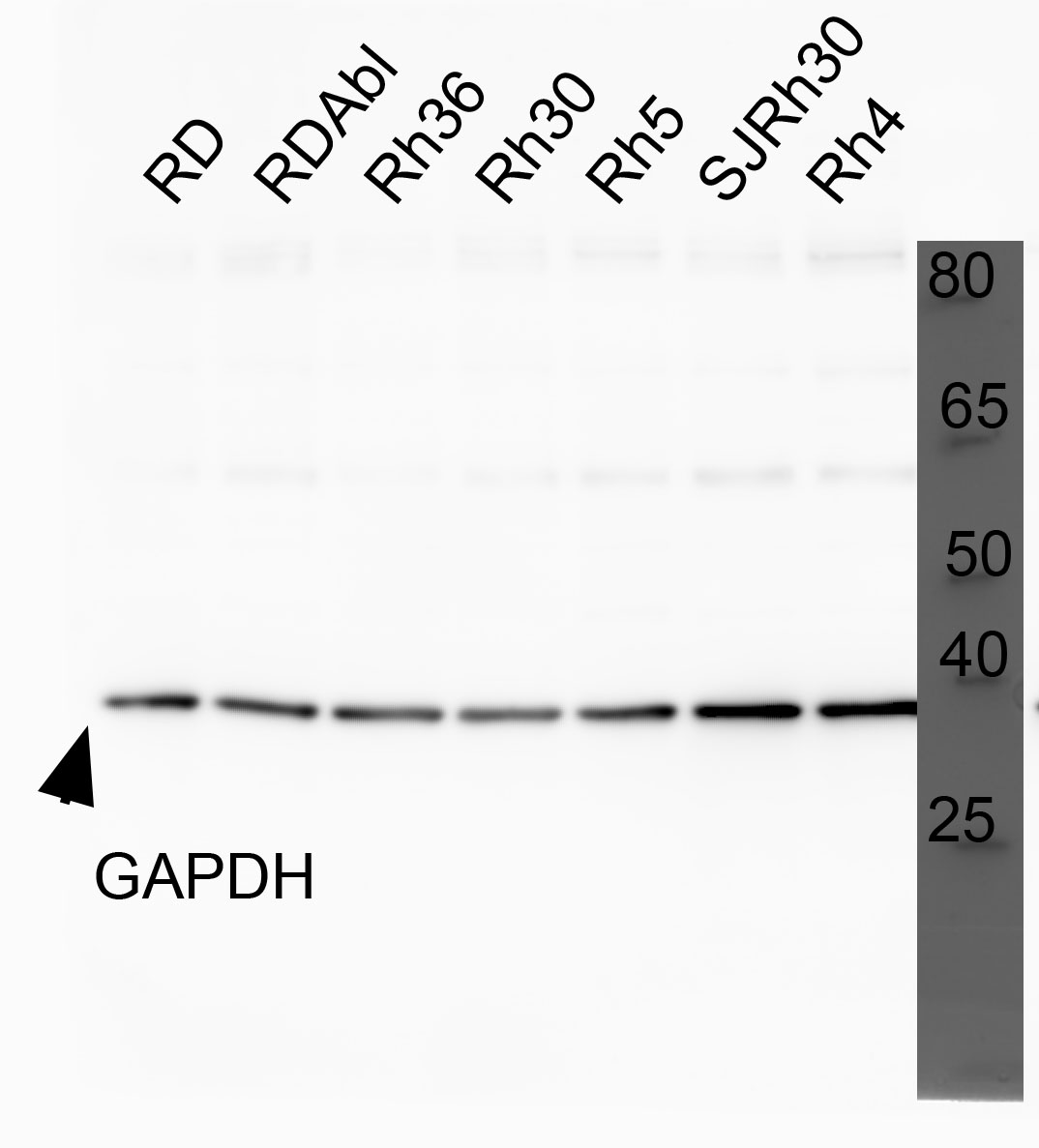

Supplement: S2 Raw image — (TIF) [file pgen.1009164.s016.tif]

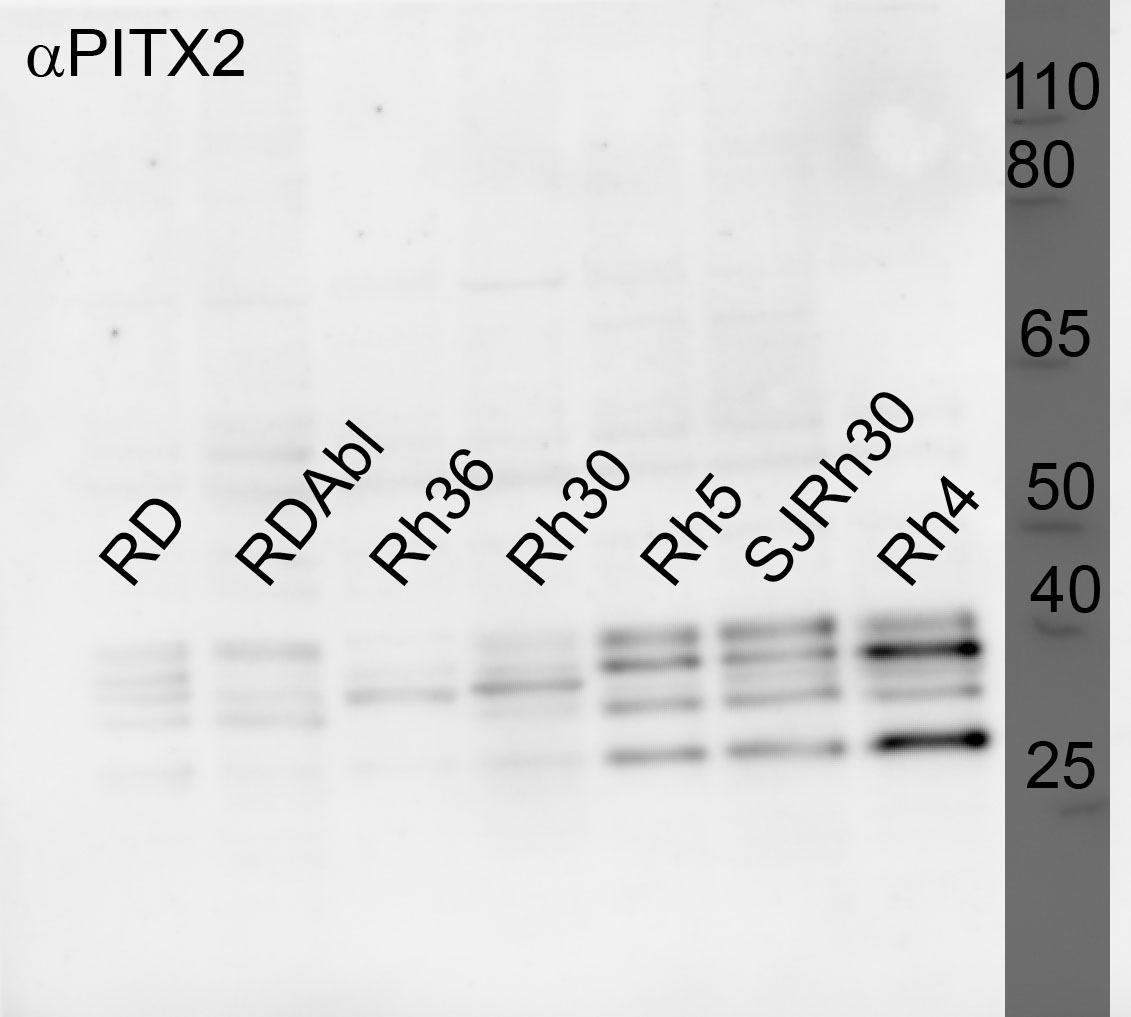

Supplement: S3 Raw image — (TIF) [file pgen.1009164.s017.tif]

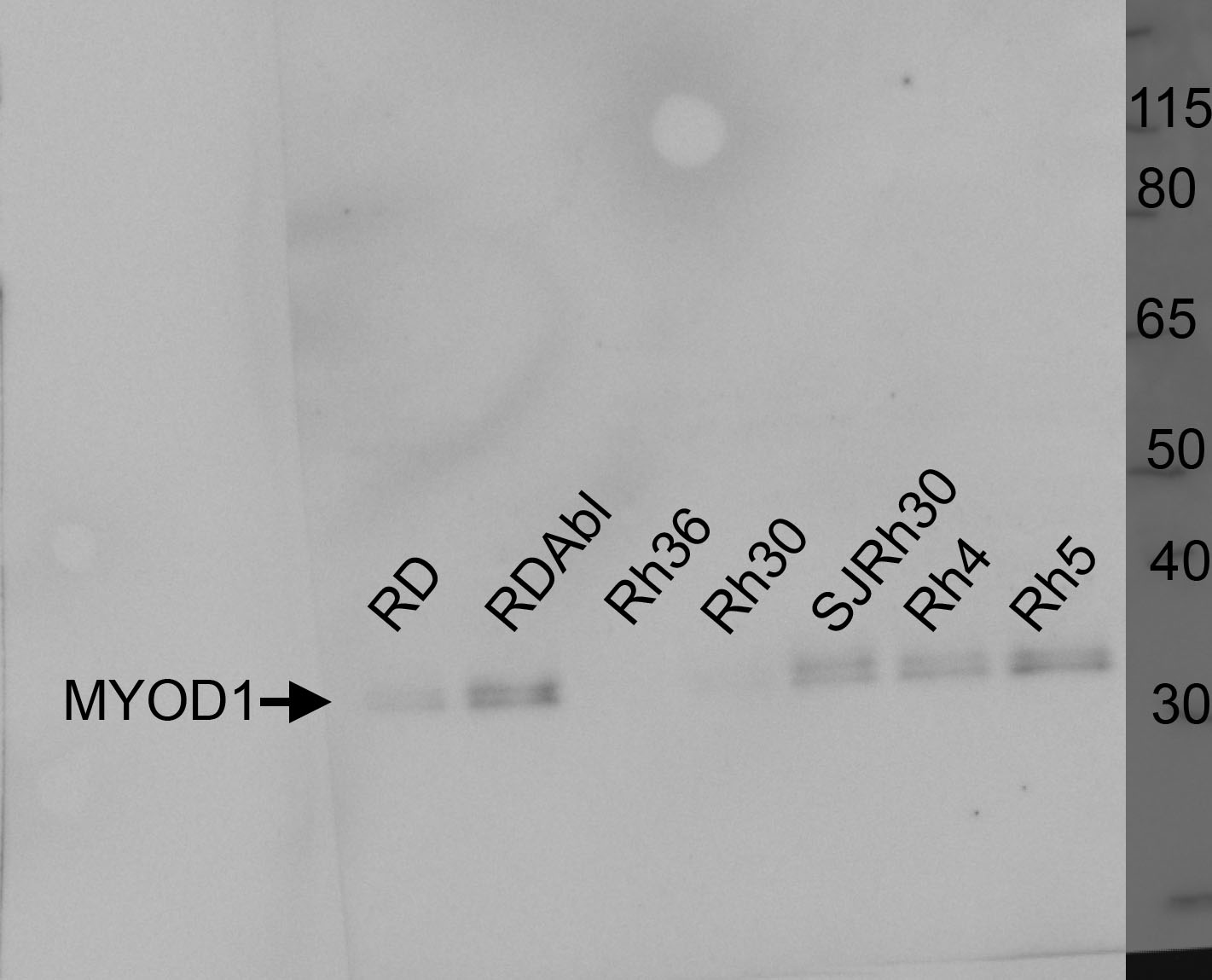

Supplement: S4 Raw image — (TIF) [file pgen.1009164.s018.tif]
