# Supplementary material for: The PAX-FOXO1s trigger fast trans-differentiation of chick embryonic neural cells into alveolar rhabdomyosarcoma with tissue invasive properties limited by S phase entry inhibition
Source: PLoS Genet. 2020 Nov 11;16(11):e1009164. doi: 10.1371/journal.pgen.1009164 (PMC7682867; doi:10.1371/journal.pgen.1009164)
Supplement: S1 Methods — (DOCX) [file pgen.1009164.s008.docx]

S1 Methods

- 1. Cloning PAX3-FOXO1 bound enhancers

Mouse versions of PAX3-FOXO1 bound CRM nearby the *Met, Meox1, Myod1* and *Alk* genes were cloned upstream of the thymidine kinase (tk) promoter and *nuclear LacZ* and using the following primers:

*Met1^CRM^-Fwd*: *TCCCAAGGCAGCTGCTACA*

*Met1^CRM^-Rev*: *TGCGCTGTTTCCAGGGATC*

*Meox1^CRM^-Fwd*: *CTCGAGGGAGTTGTTTCCT*

*Meox1^CRM^-Rev*: *GCATGCTCCCGGCCGC*

*Myod1^CRM^-Fwd*: *TCCAGAATGGGCTCGGTTC*

*Myod1^CRM^-Rev*: *ACATGGTGACAAGGAATGGC*

*Alk^CRM^-Fwd*: *TCTCCTTTTCAGCCACAGTG*

*Alk^CRM^-Rev*: *TGGCCAGCAAGCTCCTT*

Human versions of PAX3-FOXO1 bound CRM nearby the *CDH3* and *PRDM12* genes were cloned within the SacI and XhoI sites sitting upstream of *adenovirus major late* promoter (mlp) and *H2B-Turquoise* and using the following primers:

*PRDM12^CRM^-FW: GCGAGCTCCTCCACTTCCCCTTCAATGT*

*PRDM12^CRM^-Rev: CCGCTCGAGCGGCTCGTAGGACTTGAATA*

*CDH3^CRM^-FW: GCGAGCTCCCGGCTAAGGGAATGCTC*

*CDH3^CRM^-REV: CCGCTCGAGCGCTAAACATCATATCTGGCA*

- 1. Primer sequences used for RT-qPCR on chick FAC sorted neural cells

*Fw- ADAM10: CGATAATCCTGCTGTGCTCCTG*

*Rev- ADAM10: TGAAAGTCGAGGCGCAAGAAC*

*Fw- ALK: TGAGCAGTCTGGATCTCCCAA*

*Rev- ALK: TCCAGCTCACAAGGAGTCTCA*

*Fw-ARHGAP25: AGCCTGGAGTGCCTACTGAAA*

*Rev- ARHGAP5: CGTGCAAGTCCAAAGTCAGC*

*Fw-CCND1: TCCATCAGACCCGACGAGTT*

*Rev-CCND1: GGGGTCATTGCAGCCAGATT*

*Fw-CDK2: GTACAAGGCTCGCAACAAGC*

*Rev-CDK2: GAGCTTGTTCTCCGTGTGGA*

*Fw-EYA2: AGCCTGGAGTGCCTACTGAAA*

*Rev-EYA2: CGTGCAAGTCCAAAGTCAGC*

*Fw-FGFR4: GCGCAACTTCACCATCTCTGTA*

*Rev-FGFR4: AGCGTACAGCTTCTTGTCCAT*

*Fw-LMO4: ACCAAGAGCGGCATGATCC*

*Rev- LMO4: ATGATAGACATTGCCCTGTGCC*

*Fw-MEOX1: ACCTGACAAGGCTCAGGAGAT*

*Rev-MEOX1: TATGGCACTACTCGGAGCTGG*

*Fw- MYOD1: AGCCTGGAGTGCCTACTGAAA*

*Rev-MYOD1: CGTGCAAGTCCAAAGTCAGC*

*Fw-MYCN: TCTTCCCCTTCCCCGTCAA*

*Rev-MYCN: GAGCGTCTTTTCTCCACTGTCA*

*Fw-PAX2: ACCTGACGTGGTGAGACAAAG*

*Rev-PAX2: ACTACTTTGGGGGTCGCTACT*

*Fw-PITX2: AGATCGCCGTCTGGACCAA*

*Rev-PITX2: GGCTGCATCAGGCCATTGAA*

*Fw-PRDM12: CTGGTACGGGAACTCACACAAC*

*Rev-PRDM12: CACACGAAGGGCTTGTCCA*

*Fw-RB1: AACAGCGAGAGCCACGTAAA*

*Rev-RB1: TGCTTCTGCATTCTTGTTCGAG*

*Fw-SNAI1: TGTGTCTGCAAGATGTGCGG*

*Rev-SNAI1: GGAGCAGGTTTTGCACTGGT*

*Fw-TBP: TCGTGCCCGAAATGCTGAAT*

*Rev-TBP: TGCTCCTGTGCACACCATTT*

*Fw-TFAP2α: CTCTGGAAGCTGACGGATAACA*

*Rev-TFAP2α: GTCCTGAGACTGGGGGTAGAT*

- 1. Primer sequences used for qRT-PCR on FP-RMS cell lines

*Fw-ALK: TCTCATCGCAGCCGATATGG*

*Rev-ALK: GGCATCTCCTTAGAACGCTCT*

*Fw-ARHGAP25: CCTGGAGCACGGCCGGAATG*

*Rev-ARHGAP25: ACCACGGGCTCTGGGAGGTC*

*Fw-EYA2: ACCCCCAGTATTACGGCTCA*

*Rev-EYA2: TTTCGCTGGTGTGGAAGGTC*

*Fw-FGFR4: CCATAGGGACCCCTCGAATAG*

*Rev-FGFR4: CAGCGGAACTTGACGGTGT*

*Fw-FOXF1: CTCCCTGGAGCAGCCGTATC*

*Rev-FOXF1: ACTCCTTTCGGTCACACATGC*

*Fw-LMO4: GGCACGTCCTGTTACACCAA*

*Rev-LMO4: CGCCCTCATGACGAGTTCAC*

*Fw-MEOX1: GGGAGCACTGCCAATGAGAC*

*Rev-MEOX1: ATATCTGCGGAGCCGAGTCA*

*Fw-MYOD1: GAGCACTACAGCGGCGAC*

*Rev-MYOD1: TAGTAGGCGCCTTCGTAGCA*

*Fw-NHLH1: TTGAGCACCCAGAGGAGACT*

*Rev-NHLH1: CCACTTCAGGGTTCCATGGTC*

*Fw-PAX2: CTGGGGATTCCTCGCTCCAA*

*Rev-PAX2: CCACCTCCTCTAATGTGGGC*

*Fw-PAX3-FOXO1: TCCAACCCCATGAACCCC*

*Rev-PAX3-FOXO1: GCCATTTGGAAAACTGTGATCC*

*Fw-PITX2: GACTCCTTCGGAACTTGGCAC*

*Rev-PITX2: CCCAGAAGTAGCAGTTTGGCG*

*Fw-PRDM12: CACCGGAGCTGGATGACCTA*

*Rev-PRDM12: CCGTACCACACCAGCAGTTC*

*Fw-TBP: CACGAACCACGGCACTGATT*

*Rev-TBP: TTTTCTTGCTGCCAGTCTGGAC*

*Fw-TFAP2β: ATTTGAACCGGCAGCACACA*

*Rev-TFAP2 β: TGGGTCGGCTGTTCCCTATC*

**Western Blots**

FP-RMS and FN-RMS cells were lysed in a buffer composed of 10mM Tris-Cl pH7.5, 5mM EDTA, 150mM NaCl, 30mM Sodium pyrophosphate, 50mM Sodium fluoride, 10% glycerol, 1% NP40 and Complete Protease Inhibitor Cocktail (Sigma). Western blots were performed using the products and protocol by Thermo Fisher. They were revealed using the following primary antibodies: mouse anti-GAPDH (AB0067-20, Sicgen, 1:1000), mouse anti-Pitx2 (AF7388, R&D System, 1:1000), mouse anti-FOXO1 (CH-19, Cell signalling, 1:1000), rabbit anti-MYOD1 (sc-304, Santa Cruz, 1:500) and horseradish peroxydase-conjugated secondary antibodies (Jackson Immunoresearch).

- 1. List of antibodies used for immunostaining

| Antigen | Species | Dilution | Provider (reference) |
| --- | --- | --- | --- |
| ARL13b | Rabbit | 1/1000 | [1] |
| Cleaved CASPASE3 | Rabbit | 1/500 | Cell signalling (9661S) |
| CDH2 (N-Cadherin) | Mouse | 1/100 | Santa Cruz (sc-393933) |
| CDNK1c | Rabbit | 1/50 | Santa Cruz (sc-8298) |
| Activated β-CATENIN | Rabbit | 1/500 | Life Technologies (A-11132) |
| βGalactosidase | Mouse | 1/500 | Promega (Z378) |
| GFP | Chicken | 1/1000 | Abcam (ab13970) |
| Phospho- Histone H3 | Rabbit | 1/5000 | Millipore (06-570) |
| HUC/D | Mouse | 1/1000 | Thermo Fisher Scientific (16A11) |
| β1-INTEGRIN | Mouse | 1/1000 | Sigma (MAB19294) |
| LAMININ | Rabbit | 1/500 | Sigma (L9393) |
| LHX1/2 | Mouse | 1/20 | DSHB (4F2) |
| MCM2 | Rabbit | 1/500 | Ozyme (3619T) |
| PARD3 | Rabbit | 1/1000 | Millipore |
| PAX2 | Rabbit | 1/500 | Thermo Fisher Scientific (716000) |
| PAX6 | Mouse | 1/50 | DHSB (AB 528427) |
| Phosphorylated RB1 | Rabbit | 1/500 | R&D systems (MAB6495) |
| SOX2 | Rabbit | 1/200 | Thermo Fisher Scientific (48-1400) |
| TFAP2α | Rabbit | 1/100 | Santa Cruz (sc8975) |
| *Secondary antibodies*: donkey against mouse, goat, guinea pig or rabbit IgG coupled to Alexa Fluorophores A488, A546, A568 or A647 (Thermo Fisher Scientific) were diluted 1:500 and used with DAPI (500ng/ml, Sigma, 28718-90-3). | | | |

***In situ* probes**

Chick *PITX2a* isoform and *LMO4* probes were previously described [2,3]. Chick *MYOD1* probe was synthetized from PCR products generated using the following primers and cDNA prepared from HH25 chick embryos:

*Fw-cMYOD1-probe CGCCGATGACTTCTATGACG*

*Rev-cMYOD1-probe GGTAATACGACTCACTATAGGGCGGAGCAATTTGTTCTCGGT*

**Supporting References**

1. Caspary T, Larkins CE, Anderson K V. The Graded Response to Sonic Hedgehog Depends on Cilia Architecture. Developmental Cell. 2007;12: 767–778. doi:10.1016/j.devcel.2007.03.004

2. Angeles Rabadán M, Usieto S, Lavarino C, Martí E. Identification of a putative transcriptome signature common to neuroblastoma and neural crest cells. Developmental Neurobiology. 2013;73: 815–827. doi:10.1002/dneu.22099

3. Amand TRSt, Ra J, Zhang Y, Hu Y, Baber SI, Qiu M, et al. Cloning and Expression Pattern of ChickenPitx2:A New Component in the SHH Signaling Pathway Controlling Embryonic Heart Looping. Biochemical and Biophysical Research Communications. 1998;247: 100–105. doi:10.1006/bbrc.1998.8740
