# Supplementary material for: The PAX-FOXO1s trigger fast trans-differentiation of chick embryonic neural cells into alveolar rhabdomyosarcoma with tissue invasive properties limited by S phase entry inhibition
Source: PLoS Genet. 2020 Nov 11;16(11):e1009164. doi: 10.1371/journal.pgen.1009164 (PMC7682867; doi:10.1371/journal.pgen.1009164)
Supplement: Attachment — Submitted filename: Curto_response_to_referees.pdf [file pgen.1009164.s019.pdf]

Vanessa Ribes
Transcriptional network and cell fate IJM Group
IJM CNRS UMR7592, 15 rue Hélène Brion 75013 Paris
vanessa.ribes@ijm.fr
Tels: +33 (0) 1 57 27 81 94 ; +33 (0) 6 49 45 08 82

We are grateful to the referees for their thoughtful, constructive and supportive remarks. We believe they have helped us to improve and clarify our manuscript. Below we describe the new data, figures and writing changes (in blue in the text) incorporated in the new version of our paper that tackle the specific points raised by each referee.

Reviewer #1:

Major points:

1. Throughout the paper, all the figure panels should display a) examples of immunostaining; and b) quantification; for all the different conditions tested (empty vector, PAX3, PAX7, P3-FOXO1 and P7-FOXO1). Here are some specific examples:
- Figure 1: show immunostaining for the empty vector
- Figure 1: quantification (v, vi, vii) needs to be done for PAX7 and PAX7-FOXO1
- Figure 1C: No panel for PAX6 staining in the PAX7-FOXO1 cells
- Figure 2F: please show the staining for PAX3 alone (and possibly add PAX7 and PAX7-FOXO1)
- Figure S1C: top panel shows TFAP2 quantification for PAX7 and PAX7-FOXO1, but in the bottom panel PAX7 and PAX7-FOXO1 are absent from the PAX2 quantification
- Figure 3: the authors tested only one enhancer for the PAX7-FOXO1 fusion - etc…

We have followed the advices of referee 1 and 3 (see referee 3 minor point 2) and propose a new version of the manuscript with less dense figures. We have split the big figures by creating both new main and supplemental figures. We have removed all the data that were not mandatory to reach our conclusions. These comprise the distance of nuclei at 48hpe, the orientation of nuclei at 12hpe, labels of the primary cilia, profile of PARD3 at 24hpe, the activity of *hPRDM12*^{CRM} upon PAX7FOXO1 electroporation.

In addition, to "improve the clarity of the manuscript and to follow the text better", we propose a version of our study where the transformation properties of the PAXFOXO1 proteins in the neural tube are first assessed using PAX3FOXO1 (Fig 1, 3-7, S2-S6 Fig) and then compared to that of PAX7FOXO1 (Fig 8, S7 Fig). For the vast majority of the traits analysed, data in embryos electroporated with the wild-type PAX3 and PAX7 are now presented.

Within the given timeframe for the revision, we could not perform all the experiments that we had done on chick embryos electroporated with the empty vector, Pax3 and PAX3FOXO1, on embryos electroporated with Pax7 and PAX7FOXO1. Instead, we have evaluated the state of the major PAX3FOXO1 induced traits in presence of PAX7FOXO1 or the normal Pax7 (Fig 8, S7 Fig). PAX7FOXO1 transforming potential appeared highly reminiscent to that of PAX3FOXO1.

PAX7FOXO1 induced the expression of few FP-RMS signature genes at the expense of pan progenitors and post-mitotic neuronal markers (SOX2, HUC/D). Yet, for two of the FP-RMS markers assayed, *LMO4* and *PITX2a*, PAX7FOXO1 induction was less strong than that of PAX3FOXO1. This supports the idea that aspects of the transformation by PAX3FOXO1 and PAX7FOXO1 may differ[1]. We believe that further digging into this question is out of the scope of our study. Importantly, PAX7FOXO1 induces an epithelial-mesenchymal transition of the spinal progenitors and their escape from the neural epithelium and inhibits the cell cycle progression by reducing the activity of CDK, as shown for PAX3FOXO1.

We have chosen not to show images of embryos electroporated with the empty vector, as this would have further densified our figures (see the last comment of referee #3). For most markers assessed, the non-electroporated side of the neural tube or other adequate control regions within the embryos are always displayed. They are few cases where we show *pCIG* electroporated embryos or cells (Fig 4, 5), either because we use the GFP to trace cells in the 3 dimensions of the embryos or to monitor the basal activity of reporters of conserved and previously identified PAX3FOXO1 bound enhancers.

Vanessa Ribes
Transcriptional network and cell fate IJM Group
IJM CNRS UMR7592, 15 rue Hélène Brion 75013 Paris
vanessa.ribes@ijm.fr
Tels: +33 (0) 1 57 27 81 94 ; +33 (0) 6 49 45 08 82

2. The authors elegantly combined and re-analyzed microarrays from different ARMS studies in Table S1 and S2, which provide a rich resource for anyone aiming at identifying key molecular events taking place in these tumors. However, in Figure 2, the authors opted to focus on a set of transcription factors which expression is elevated in ARMS. Are these TFs the best and more relevant markers for ARMS? What about the set of genes that are normally exclusively expressed in the myogenic lineage (in addition to MYOD)? Or what about the genes that display the highest up-regulation in ARMS, irrespective of their function?

To analyse the phenotype of PAXFOXO1$^+$ cells in the chick embryos we needed to work with a limited number of FP-RMS signature genes that we had redefined by combining the transcriptomic data from 192 patients. This was to be able to assess i) where were the cells that responded to the PAXFOXO1s in the embryonic neural tissue (*In situ* hybridization and fluorescent immuno-labelling), quantify the induction levels of genes (RT-qPCR and quantification of fluorescent immuno-labelling), or assess cell to cell variability in the response of cells (measures of fluorescent signal within single cells).

We focused on TFs, as they are well recognized regulators of cell state [2]. This is mediated notably by their ability to promote or repress gene expression. We chose TFs that were known to impact on cell identities and differentiation in embryonic lineages as they have been shown to be major players in the establishment of dysfunctional transcriptomic state in cancer cells[2], and recently in FP-RMS cells[3,4]. Amongst the 1194 genes enriched in FP-RMS in comparison to FN-RMS, the TFs we chose are all in the vicinity of previously identified PAX3FOXO1 binding sites[3,5] and some of them were shown to be sensitive to PAX transcriptional activity in the neural tube (PRDM12 for instance; [6]). More importantly, we chose TFs that allow us to distinguish PAXFOXO1 transformed cells from any other cells in the embryo, thus we chose TFs which are not co-expressed in the embryo, notably TFs which will not mark muscle cells. Finally, we validated that these TFs were indeed good FP-RMS markers. We have incorporated these explanations in our result and discussion sections.

Regarding the ability of PAX3FOXO1 to induce other FP-RMS specific genes, which are not TFs, we provided data already in our previous version of our manuscript for *ARHGAP15, ALK, FGFR4*. All of these are induced by PAX3FOXO1 in the chick neural tube and induced to a much lesser extent by PAX3.

The core bHLH transcriptional cascade that paces the tempo of myogenesis is rough terms structured as follow MYF5→MYOD1→MYOG[7]. MYF5 as opposed to MYOD1 and MYOG is not enriched in FP-RMS compared to FN-RMS (S1-3 tables), hence we haven't investigated its status in the chick PAXFOXO1 expressing cells. For MYOD1 see referee 1 minor point 9. We checked for MYOG status by performing immunostaining at 48hpe in PAX3 and PAX3FOXO1 samples. In neither samples, it is induced (S2B Fig). This can be explained by the fact that MYOG does not stand as one of the putative PAXFOXO1 direct targets. It is not in the vicinity of previously identified PAX3FOXO1 binding sites (S3 table). It is worth mentioning that although MYOG stands as one of the best molecular markers for RMS samples, its expression displays inter-patient's heterogeneity [8]. Whether this variation stem from tissue specific response to PAX3FOXO1 remains to be assessed (several cellular subtypes have been proposed to contribute to FP-RMS). Alternatively, in the light of the Maroto et al., 1997's paper (brought to us by the referee 3), one can propose it takes more than 2 days to be able to see the myogenic cascade to be implemented in PAX3FOXO1's cells.

Minor points:

1. Does the expression of PAX3-FOXO1 or PAX7-FOXO1 drive oncogene-induced senescence? It may be out of the scope of the study, but that would strengthen the paper.
We had also been very enthusiastic regarding this hypothesis. However, several lines of evidence went against it.

Vanessa Ribes
Transcriptional network and cell fate IJM Group
IJM CNRS UMR7592, 15 rue Hélène Brion 75013 Paris
vanessa.ribes@ijm.fr
Tels: +33 (0) 1 57 27 81 94 ; +33 (0) 6 49 45 08 82

(i) Senescent cells normally display undetectable levels of the phosphorylated form of RB1 (pRB1). Unphosphorylated RB1 is very active and inhibits the activity of the replication E2F TFs [9]. This RB1 hyperactivity is due to the elevated expression and activity of CDK inhibitors, such as p16 (CDKN2A) and/or p53. PAX3FOXO1 and PAX7FOXO1^{+} chick neural cells displayed reduced levels of pRB1, but not extinguished pRB1. To illustrate this, we have now quantified pRB1 levels in cells that have left the cell cycle in the developing neural tube, i.e. the post-mitotic neurons (Fig 6Diii). The data shows that the levels of pRB1 are intermediate between pRB1 levels in the proliferating spinal progenitors and differentiated neurons. Hence, CDK activity is lowered down by the fusion TFs but not completely abolished. Accordingly, while senescent cells are no longer able to enter S phase, PAX3FOXO1^{+} cells were capable of incorporating EdU, yet less efficiently than control cells (Fig 6B).

(ii) We have performed senescence-associated beta-galactosidase assays (SA-βGal, see referee Fig 1); SA-βGal being a landmark of senescent cells (e.g.[10]). These assays showed that the floor plate cells in the neural tube or the apical ectodermal ridge of the limb bud were positive for SA-βGal as expected [10]. In contrast, cells electroporated with either Pax3, Pax7, PAX3FOXO1 or PAX7FOXO1 were not positive for SA-βGal, further indicating that the gain for the PAX variants does not bring cells towards senescence.

(iii) In agreement with this, a previous study on the cross-interactions between PAX3FOXO1 activity and the senescence regulator p16 has shown that PAX3FOXO1 activity rather block p16 activity, by reducing its expression [11]. Furthermore, primary human skeletal muscle cells are able to bypass senescence in presence of PAX3FOXO1.

Altogether, this has brought us to revise the words used to describe the slowing down of the cycling activity of PAXFOXO1^{+} cells.

2. In Figure 2, the authors should disclose which proportion of the ARMS samples display PAX3-FOXO1 fusion, PAX7-FOXO1 fusion or no fusion. This information can be found in Supplemental Table S3, but it should appear in the manuscript.

We have generated a new heatmap where the biopsies carrying either PAX3FOXO1 and PAX7FOXO1 are visible (new Fig 2A). In the result section, we now state: "*we combined and re-analysed microarray-based tumour transcriptomes obtained from 99 PAX3FOXO1 and 34 PAX7FOXO1 positive FP-RMS patients and 59 patients affected by other RMS subtypes*".

3. In Figure 2, the authors analyze the expression of EYA2, but its relevance is not specified in the text. Please edit and add the appropriate references.

We thank the referee for spotting this. *EYA2* expression is reminiscent to that of *LMO4* both of which are present in both the embryonic muscle cells and the NCC derived peripheral nervous system upon somitogenesis. Because both the expression patterns and the function of EYA2 has determined our choice towards this TF, we cite a functional study on EYA2 in the embryo [12].

4. In Figures 2Dv and 2Ev, the authors should indicate which genes are statistically and significantly different in ARMS (2Dv) and in PAX3-FOXO1 (2Ev), for example by using an asterisk.

This has been modified accordingly. See new Fig 2E and Fig3B. We have also extended the description of the figure.

5. In Figure 2E, it would be interesting and relevant to determine whether these sets of genes are also elevated in PAX7-FOXO1 fusion.

See major point 1. We had focused on 4 of these genes, *LMO4, PITX2a,* PAX2, TFAP2α (See Fig 8 and S7 Fig). Yet and as said earlier, the straight comparison between the transcriptional potential of PAX3FOXO1 and PAX7FOXO1 are out of the scope of this study, although key to further understand inter-patient heterogeneity and outcome[1].

Vanessa Ribes
Transcriptional network and cell fate IJM Group
IJM CNRS UMR7592, 15 rue Hélène Brion 75013 Paris
vanessa.ribes@ijm.fr
Tels: +33 (0) 1 57 27 81 94 ; +33 (0) 6 49 45 08 82

6. In Figure S1B, the authors should perform additional immunoblottings for the different genes they highlight in Figure 2, in addition to PITX2. These additional Western blots should include MYOD.
This is now provided in the new version of S1B Fig. It is detected in 2 out 3 FN-RMS and in all FP-RMS cell lines, yet weakly detected in Rh30 cells.

7. It looks like throughout the paper, the statistics are calculated using a n number that corresponds to the number of cells analyzed. The n number employed for statistical tests should correspond to the number of biological replicates (i.e. number of embryos).
We apologize for the lack of precision in the first version of the manuscript. We have revised all our figure legends and the methods section to be consistent with the treatment of the data described below.

Regarding data in the chick embryo, the n in the figure legend are always referring to the number of embryos analysed. To avoid confusion, we changed the "n=" in previous figure 4Eiv for "# of cells =" in the new Fig 5Civ.

Regarding the statistical treatments and representation of the data generated in chick embryos.
i) The % of SOX2^{+}, HUC/D^{+}, PH3^{+}, EDU^{+}, activated CASPASE3^{+} cells in the GFP^{+} population, have been evaluated on between 2 to 6 transverse sections per embryo. The number of sections taken into account per embryo varies as a function to the extent of electroporated cells found along the anterior-posterior axis of the embryo. The mean value per embryo has been calculated and is now represented as a dot on the graphs shown in Fig 1Biii,vi; 6Aiii; 6Biii; 7Aiii; Bii; S5Aiv, S5Biv; S5D, S6Aiii,v; S7Aiii,vi; S7C). The probability of similarity between two populations of embryo mean values (i.e. between two types of chick samples) were evaluated using a Mann-Whitney U Test. This non-parametric test overcomes the assumption of sample normality. The size of the populations tested varied between 3 to 10 embryos per population.
ii) For many parameters, such as the levels of reporter activity or the levels of expression of pRB1, our plots display single cell values (see for example Fig 4vii). These values were measured at least in 3 embryos and often in more than 5 embryos, the number of embryos analysed is always given in the figure legend. The probability of similarity between two populations of cells comprising cells of several embryos using a Mann-Whitney U Test. We have validated that the greatest variations in our data are set between cells and not between embryos. This is likely due to the developmental stage of each cells, their localisation within the neural tube and the levels of expression of the PAX variants. We would like to add that two-way anova is not implementable because of the great variations in the number of cells measured per embryos, the variability due to their position….etc… Split-plots are not reader-friendly.

8. In the text, the authors state that "Conversely, forced expression of PAX3 or PAX7 tented to reduce the number of TFAP2+ and PAX2+ neurons generated ». This is not true for TFAP2. As for PAX2, the data is not shown for the PAX7 condition. For these reasons, the authors statement is not justified.
Our statement now matches our observations and quantifications.

9. In Figure 2F, the authors should add a staining for MYOD, a gene that is normally expressed exclusively in myogenic cells, unlike LMO4 and PITX2.
We have tried several antibodies against MYOD1, none of them could detect the chick version of MYOD1. We thus generated *MYOD1* ISH probe.
While we previously showed that the expression of this gene was increased at 48hpe by RT-qPCR, our *in situ* data shows that actually *MYOD1* expression can be detected in PAX3FOXO1 samples 24h earlier. It is interesting also to note that while other FP-RMS markers (*LMO4*; *PITX2A*) were induced in most cells, that of *MYOD1* was restricted to only part of the cells. These results are now discussed see also response to referee#3.

10. It looks like the same dataset is presented in Figure 5Aiii and S3Av. Please make the appropriate corrections in the revised manuscript.

Vanessa Ribes
Transcriptional network and cell fate IJM Group
IJM CNRS UMR7592, 15 rue Hélène Brion 75013 Paris
vanessa.ribes@ijm.fr
Tels: +33 (0) 1 57 27 81 94 ; +33 (0) 6 49 45 08 82

In the new version of the paper, we have avoided this, but in the graph Fig 7Aiii, which displays the data shown in Fig 6Aiii and the graphs in Fig8C which display values in the *pCIG* shown in Fig 6. We thought that they serve as a base line and allow to present the statistical analysis regarding the differences observed. The redundancy is clearly stated in the figure legends.

11. In Figure 5B, please specify which plasmid was electroporated in the embryo from which the representative immunostaining picture was selected (5Bi and 5Bii).
This has been corrected.

12. In Figure 5C, the authors should include a representative flow cytometry plot (cell cycle) for all the different conditions. In addition, it is not clear in the Methods section how the proportions of cells in the different cell cycle phases was calculated. Separating the flow cytometry plot into 3 bins is not appropriate to assess the distribution into cell cycle phases. Many softwares are available to analyze cell cycle profiles, for example ModFit LT. The authors should perform cell cycle analyses with an appropriate software and clearly indicate it in the Methods section.
We've rectified our oversight and modified our Methods Section. One can now read "*Fluorescent intensities were quantified using a Cyan ADP Beckman Coulter analyser and cell cycle phases determined using Summit software (Beckman Coulter).*"

To avoid increasing the density of figures (see Minor point 2, referee #3), we are showing the flow cytometry plots in a new supplemental figure (S6A Fig).

Reviewer #2:
We thank the reviewer for the very positive comment.
There are a few typos in the manuscript that the authors should correct. For example page 9 "Conversely, forced expression of PAX3 or PAX7 tented to reduce …" should be "tended to reduce.".
We have hopefully removed most of the typos across the text.

Reviewer #3:
Major Considerations:
Of note, both of these studies are not referenced in the manuscript, which is a very important missing component. In tandem, these two studies have already reported the main selling-points to this manuscript's primary potential impact. Thus, even though the authors build nicely on these previous models given modern-day technology, the manuscript reads more as a modern-day extension rather than a novel study in the RMS field.
We are grateful to the referee for bringing up these two studies. We apologize for the initial miss-out. Yet, we wish to highlight that we did cite the original paper presenting the model used in Finckenstein et al, Transgenic Res (2006).

The data presented in these two studies are not redundant with ours and do not question the novelty of our study. Rather they call to discuss the questions of i) what are the necessary parameters for the PAX variants to induce myogenic traits in a lineage distant from the mesodermal one, notably the embryonic spinal cord? and ii) what are the arguments in favour of a transformation of PAXFOXO1+ chick neural cells into a FP-RMS like cells, can we exclude a routing towards myogenesis?

Below we compare our data to these two studies and some of the elements written below can now be found in our new discussion.

1) My only "Major Consideration" for this manuscript is that the ability of PAX3 and/or PAX3-FOXO1 to transform chick neural progenitor tissue into muscle is not unique, as at least two such published manuscripts dated ~15-25 years ago report similar findings. In Maroto et al, Cell (1997), the authors elegantly used avian neural tube explant primordia to show that ectopic wild-type PAX3 transforms

Vanessa Ribes
Transcriptional network and cell fate IJM Group
IJM CNRS UMR7592, 15 rue Hélène Brion 75013 Paris
vanessa.ribes@ijm.fr
Tels: +33 (0) 1 57 27 81 94 ; +33 (0) 6 49 45 08 82

these non-muscle precursors into muscle-lineage cells. Thus, the notion that the PAX3 DNA-binding domains can drive ectopic myogenesis in avian neural precursors is known.
There are a clear discrepancy between our data and that presented in the [13] paper. Maroto et al., show that the forced expression of Pax3 in chick neural tube explants using RCAS based viral infection can induce after 5 days *MYOD1*, *MYF5* and *MYOG* [13]. In our hands, Pax3 forced expression using electroporation failed to induce *MYOD1* in the neural tube of chick embryos (Fig 3). However, the gain for PAX3FOXO1 induce an ectopic expression of *MYOD1* in the embryonic spinal cord (Fig 3). Furthermore, our new S2B Fig shows that neither the electroporation of *Pax3* nor that of *PAX3FOXO1* is sufficient to induce the expression of the muscle differentiation determinant MYOG within 48h.

Our study and that of Maroto et al. use similar model system. They have isolated and infected chick neural explants at the developmental stage at which we have electroporated our chick embryos. Yet, we have used two distinct strategies to promote the expression of PAX variants. The RCAS viral infection used in the Maroto et al. paper allowed the integration of the transgene into the cell genome and hence to expose cells to high levels of transgene expression in a prolonged manner. On the contrary, electroporated plasmids are mainly "episomally" transcribed and get progressively diluted with cell divisions, leading to the progressive decrease in transgene product levels (e.g.[14]). This argues for the levels of PAX3 to be determinant in the acquisition of myogenic traits by neural cells.

Sustaining this idea of the importance of PAX variants levels, PAXFOXO1 tumorigenic activity relies on high levels of PAXFOXO1 TFs [15–17]. On the same line, it has long been recognized that the levels of wild-type PAX have strong impact on cell fate during development. The best example of this stands in the strong NCC developmental defects caused by the loss of one single PAX3 allele (cf. the human Waardenburg syndrome [18] or splotch mice phenotype). We have also shown that playing with the number of PAX3 and PAX7 alleles generates discrete combination of neuronal subtypes in the spinal cord [6]. More importantly, we have observed in our study and described that spinal progenitors displayed PAX3 dose dependent responses. While low levels of PAX3 expression does not change the expression levels of the pan-neurogenic markers SOX2 and PAX6, the levels of these two proteins are decreased when cells are submitted to high levels of PAX3 (Fig 1Bii, Ci', S7Ai'). This could be indicative of a transition in their transcriptomic state. Explaining why these cells expressing high levels of PAX3 did not induce MYOD1 could stem from a requirement of a long exposure to PAX3 activity (48h in our study against 5days in the Maroto et al.'s study).

This then raises the question of why high levels or prolong exposure to PAX3 would be required for spinal cells, which already express endogenously some PAX3, to express myogenic markers. Many studies on tissue specific action of TFs have contributed to the idea that this holds to the presence of specific co-factors or proteins which modulates the transcriptional potential of the TFs (Post-translational regulation, such as the phosphorylation). Whether such PAX3 specific cofactors or co-modulators exist in the neural and/or myogenic lineage remains to be fully studied. Yet, several lines of evidence suggest that the transcriptional potential of PAX3 differs in these two lineages. In the myogenic lineage PAX3 acts mainly as a transcriptional activator. As such its recruitment to the genome is associated positive chromatin marks [19] and Pax3 loss of function phenotypes can be rescued by the expression of PAX3FOXO1 [20]. Conversely, in the neural tube it would act at least partially as a transcriptional repressor [6], PAX3FOXO1 does not rescue Pax3 loss of function phenotypes [6]. Even more it can interfere with the normal function of PAX3 and leads to specific cell fate changes ([6,16,21] and our study). In agreement, PAX3 poorly induced the activity of FP-RMS in the neural tube of chick embryos (Fig 4, S2B Fig). Interestingly, it has been observed that the fusion with FOXO1 inhibits this repressive activity [22] and therefore the PAX3FOXO1 proteins harbour a higher transcriptional potential, which certainly relies less than PAX3 on the tissue specific co-factors. In agreement, known co-factors of the PAX3FOXO1, such as BRD4, are ubiquitously expressed ([3]; http://www.informatics.jax.org/image/MGI:3498125). How the levels of PAX3 could help bypassing a repressive activity and favour a positive one remains to be studied. To speculate, increasing the levels

Vanessa Ribes
Transcriptional network and cell fate IJM Group
IJM CNRS UMR7592, 15 rue Hélène Brion 75013 Paris
vanessa.ribes@ijm.fr
Tels: +33 (0) 1 57 27 81 94 ; +33 (0) 6 49 45 08 82

of PAX could favour homo or heterodimerisation, which in turn could promote the recruitment to specific CRM regions. Alternatively, increasing levels and prolong duration of PAX would facilitate the establishment of feedforward regulatory loops to trigger the myogenic program. For instance, MYOD1 induction would be required to get some MYOG…etc…

In Finckenstein et al, Transgenic Res (2006), the authors report that transgenic mice expressing PAX3-FOXOI show multiple defects in muscle development, including ectopic myogenesis in the embryonic neural tube.
The paper by [21] shows that in mice carrying a transgene expressing PAX3FOXO1 using Pax3 promoter region some muscles can be formed in the ventral part of the neural tube of E17.5 mouse embryos (NB in the ventral neural tube Pax3 regulatory regions are normally not active). Before comparing this result to our data, we would like to point out elements which compromise a straight comparison of their data with ours. This is the reason why in the first version of the manuscript we did mention this study, but only the primary study ([23]). In their experimental design they could not formerly prove that these muscles are formed from the neural tube cells nor that PAX3FOXO1 was actually present in the neural tube of these mice. We quote their discussion *"In E13.5 and 15.5 embryos, however, we did not detect desmin staining within this region of the neural tube, and there were no obvious MYOD or c-met expressing cells migrating in this region"..... "Unfortunately, our inability to detect PAX3-FKHR by sectioned in situ hybridization in these stage embryos did not allow us to rule out whether PAX3-FKHR was simply mis-expressed in this ventral region".*

More interestingly, the paper raises the question of whether in our system PAX3FOXO1 induces the transdifferentiation of spinal cells into muscle cells or in FP-RMS like cells. We believe that the characterization of the profile of expression of 14 genes which are normally not expressed together in the embryos and not in muscle cells and that are all induced by PAX3FOXO1 provides a strong argument for PAX3FOXO1 to drive cells towards a FP-RMS like state, rather than a conventional embryonic muscle like state. In addition, the histology of the cells transformed by PAX3FOXO1 do not resemble that of embryonic muscle cells, we haven't uncovered any fibres like structures, for instance. Instead, the round nuclei, the mesenchymal like shape of cells is reminiscent to that of FP-RMS biopsies [24].

So we suggest that the reason why in the Finckenstein paper [21], no tumours were formed whereas in our system yes is due to of a lack of sufficient levels of PAX3FOXO1 expression in the mouse transgenesis model [6,16,21].

This reviewer believes that the authors need to explain in the manuscript how their studies are significantly novel in light of these prior studies. A convincing discussion on the issue could alter my perception.
We have remodel our discussion. We would like also here to further clarify the advances brought by our study and the perspectives it offers:
i) We have redefined the molecular identity of FP-RMS and have shown that their transcriptome not only relate to that of muscle cells, but also to that of other embryonic lineages. We have validated the use of few TFs as markers of this complex signature and have demonstrated that PAXFOXO1s, notably PAX3FOXO1, can induce these TFs. Notably, out of the nine TFs we have focused on, PITX2 protein distribution across RMS samples suggests it could be a biomarker of FP-RMS. Not only it would be a good biomarker, but in the light of the enrichment of FP-RMS super enhancers for DNA motifs known to be bound by PITX3, a paralog to PITX2[3,4] this TFs once induced could cooperate with PAX3FOXO1 to remodel the transcriptomic state of cells.
ii) Association clinical studies have shown that the malignancy of FP-RMS is greater than that of FN-RMS, and the presence of PAX3FOXO1 and PAX7FOXO1 is predictive of a still too poor patients' outcome [25]. Yet, our knowledge on how PAXFOXO1 TFs regulate tissue invasion and migration is still incomplete. Work on downstream targets of PAX3FOXO1 have revealed the implication of several cell surface protein in providing FP-RMS cells with migration properties [26–30]. We are still far for

Vanessa Ribes
Transcriptional network and cell fate IJM Group
IJM CNRS UMR7592, 15 rue Hélène Brion 75013 Paris
vanessa.ribes@ijm.fr
Tels: +33 (0) 1 57 27 81 94 ; +33 (0) 6 49 45 08 82

understanding how metastasis could be targeted. We believe that our study presents a unique model to study PAXFOXO1 based metastasis and tissue invasion. None of the numerous existing models offer this opportunity. Even mouse and zebrafish based models haven't allowed to follow up the transformation of cells and pinning the cell at the origin of the tumour [16,21]. We are now in good position to dissect the genetic-phenotypic map of metastasis and its regulation by the fusion TFs.

iii) Finally, we found that PAXFOXO1s mediated inhibition of cell cycle can be recapitulated in other cell types and overcome by activation of CDK-CYCLIN activity or by providing cells with high levels of MYCN. This offers a unique explanation for why additional mutations in cell cycle regulators increase the tumorigenic potential of PAXFOXO1s expressing cells [15–17,31–34].

I do want to restate, however, that I really enjoyed reading this manuscript and note the excellent quality of the data, especially the tissue fluorescence studies.
Thank you.

Minor Considerations:

1) Of note, the RMS field has essentially ceased using the term Alveolar RMS or Embryonal RMS. Instead, the WHO classifications are now Fusion-Positive (PAX3- or PAX7-FOXO1-positive) RMS and Fusion-Negative RMS.
We agree that this is important to employ conventional terms. We have changed the text accordingly.

2) Though the data and figures are of high quality, the figures are very, very dense to examine and process. The authors are advised to contemplate more reader-friendly configurations for their copious amount of data.
We thank the referee for encouraging at remodelling our figures, they are less dense and we believe the paper has gained in intelligibility. The new changes are described in our response to Referee#1's major point 1.

References

1. Sorensen PHB, Lynch JC, Qualman SJ, Tirabosco R, Lim JF, Maurer HM, et al. PAX3-FKHR and PAX7-FKHR Gene Fusions Are Prognostic Indicators in Alveolar Rhabdomyosarcoma: A Report From the Children's Oncology Group. JCO. 2002;20: 2672–2679. doi:10.1200/JCO.2002.03.137

2. Bradner JE, Hnisz D, Young RA. Transcriptional Addiction in Cancer. Cell. 2017;168: 629–643. doi:10.1016/j.cell.2016.12.013

3. Gryder BE, Yohe ME, Chou H-C, Zhang X, Marques J, Wachtel M, et al. PAX3–FOXO1 Establishes Myogenic Super Enhancers and Confers BET Bromodomain Vulnerability. Cancer Discov. 2017;7: 884–899. doi:10.1158/2159-8290.CD-16-1297

4. Gryder BE, Pomella S, Sayers C, Wu XS, Song Y, Chiarella AM, et al. Histone hyperacetylation disrupts core gene regulatory architecture in rhabdomyosarcoma. Nat Genet. 2019;51: 1714–1722. doi:10.1038/s41588-019-0534-4

5. Cao L, Yu Y, Bilke S, Walker RL, Mayeenuddin LH, Azorsa DO, et al. Genome-Wide Identification of PAX3-FKHR Binding Sites in Rhabdomyosarcoma Reveals Candidate Target Genes Important for Development and Cancer. Cancer Research. 2010;70: 6497–6508. doi:10.1158/0008-5472.CAN-10-0582

Vanessa Ribes
Transcriptional network and cell fate IJM Group
IJM CNRS UMR7592, 15 rue Hélène Brion 75013 Paris
vanessa.ribes@ijm.fr
Tels: +33 (0) 1 57 27 81 94 ; +33 (0) 6 49 45 08 82

6. Gard C, Gonzalez Curto G, Frarma YE-M, Chollet E, Duval N, Auzié V, et al. Pax3- and Pax7-mediated Dbx1 regulation orchestrates the patterning of intermediate spinal interneurons. Developmental Biology. 2017;432: 24–33. doi:10.1016/j.ydbio.2017.06.014

7. Buckingham M, Bajard L, Chang T, Daubas P, Hadchouel J, Meilhac S, et al. The formation of skeletal muscle: from somite to limb. J Anatomy. 2003;202: 59–68. doi:10.1046/j.1469-7580.2003.00139.x

8. Heerema-McKenney A, Wijnaendts LCD, Pulliam JF, Lopez-Terrada D, McKenney JK, Zhu S, et al. Diffuse Myogenin Expression by Immunohistochemistry is an Independent Marker of Poor Survival in Pediatric Rhabdomyosarcoma: A Tissue Microarray Study of 71 Primary Tumors Including Correlation With Molecular Phenotype. The American Journal of Surgical Pathology. 2008;32: 1513–1522. doi:10.1097/PAS.0b013e31817a909a

9. Narita M, Nuñez S, Heard E, Narita M, Lin AW, Hearn SA, et al. Rb-Mediated Heterochromatin Formation and Silencing of E2F Target Genes during Cellular Senescence. Cell. 2003;113: 703–716. doi:10.1016/S0092-8674(03)00401-X

10. Storer M, Mas A, Robert-Moreno A, Pecoraro M, Ortells MC, Di Giacomo V, et al. Senescence Is a Developmental Mechanism that Contributes to Embryonic Growth and Patterning. Cell. 2013;155: 1119–1130. doi:10.1016/j.cell.2013.10.041

11. Linardic CM, Naini S, Herndon JE, Kesserwan C, Qualman SJ, Counter CM. The *PAX3-FKHR* Fusion Gene of Rhabdomyosarcoma Cooperates with Loss of p16 ^{INK4A} to Promote Bypass of Cellular Senescence. Cancer Res. 2007;67: 6691–6699. doi:10.1158/0008-5472.CAN-06-3210

12. Grifone R, Demignon J, Giordani J, Niro C, Souil E, Bertin F, et al. Eya1 and Eya2 proteins are required for hypaxial somitic myogenesis in the mouse embryo. Developmental Biology. 2007;302: 602–616. doi:10.1016/j.ydbio.2006.08.059

13. Maroto M, Reshef R, Münsterberg AE, Koester S, Goulding M, Lassar AB. Ectopic Pax-3 Activates MyoD and Myf-5 Expression in Embryonic Mesoderm and Neural Tissue. Cell. 1997;89: 139–148. doi:10.1016/S0092-8674(00)80190-7

14. Kumamoto T, Maurinot F, Barry R, Vaslin C, Vandormael-Pournin S, Le M, et al. Direct readout of neural stem cell transgenesis with an integration-coupled gene expression switch. Developmental Biology; 2019 Nov. doi:10.1101/834424

15. Keller C, Arenkiel BR, Coffin CM, El-Bardeesy N, DePinho RA, Capecchi MR. Alveolar rhabdomyosarcomas in conditional Pax3:Fkhr mice: cooperativity of Ink4a/ARF and Trp53 loss of function. Genes & Development. 2004;18: 2614–2626. doi:10.1101/gad.1244004

16. Kendall GC, Watson S, Xu L, LaVigne CA, Murchison W, Rakheja D, et al. PAX3-FOXO1 transgenic zebrafish models identify HES3 as a mediator of rhabdomyosarcoma tumorigenesis. eLife. 2018;7: 1–28. doi:10.7554/eLife.33800

17. Ren YX, Finckenstein FG, Abdueva DA, Shahbazian V, Chung B, Weinberg KI, et al. Mouse mesenchymal stem cells expressing PAX-FKHR form alveolar rhabdomyosarcomas by cooperating with secondary mutations. Cancer Research. 2008;68: 6587–6597. doi:10.1158/0008-5472.CAN-08-0859

18. Boudjadi S, Chatterjee B, Sun W, Vemu P, Barr FG. The expression and function of PAX3 in development and disease. Gene. 2018;666: 145–157. doi:10.1016/j.gene.2018.04.087

Vanessa Ribes
Transcriptional network and cell fate IJM Group
IJM CNRS UMR7592, 15 rue Héléne Brion 75013 Paris
vanessa.ribes@ijm.fr
Tels: +33 (0) 1 57 27 81 94 ; +33 (0) 6 49 45 08 82

19. Magli A, Baik J, Pota P, Cordero CO, Kwak I-Y, Garry DJ, et al. Pax3 cooperates with Ldb1 to direct local chromosome architecture during myogenic lineage specification. Nat Commun. 2019;10: 2316. doi:10.1038/s41467-019-10318-6

20. Relaix F. The transcriptional activator PAX3-FKHR rescues the defects of Pax3 mutant mice but induces a myogenic gain-of-function phenotype with ligand-independent activation of Met signaling in vivo. Genes & Development. 2003;17: 2950–2965. doi:10.1101/gad.281203

21. Finckenstein FG, Davicioni E, Osborn KG, Cavenee WK, Arden KC, Anderson MJ. Transgenic mice expressing PAX3-FKHR have multiple defects in muscle development, including ectopic skeletal myogenesis in the developing neural tube. Transgenic Res. 2006;15: 595–614. doi:10.1007/s11248-006-9011-9

22. Bennicelli JL, Advani S, Schäfer BW, Barr FG. PAX3 and PAX7 exhibit conserved cis-acting transcription repression domains and utilize a common gain of function mechanism in alveolar rhabdomyosarcoma. Oncogene. 1999;18: 4348–4356. doi:10.1038/sj.onc.1202812

23. Anderson MJ, Shelton GD, Cavenee WK, Arden KC. Embryonic expression of the tumor-associated PAX3-FKHR fusion protein interferes with the developmental functions of Pax3. Proceedings of the National Academy of Sciences. 2001;98: 1589–1594. doi:10.1073/pnas.98.4.1589

24. Arnold MA, Anderson JR, Gastier-Foster JM, Barr FG, Skapek SX, Hawkins DS, et al. Histology, Fusion Status, and Outcome in Alveolar Rhabdomyosarcoma With Low-Risk Clinical Features: A Report From the Children's Oncology Group: Outcomes in Low-Risk Alveolar Rhabdomyosarcoma. Pediatr Blood Cancer. 2016;63: 634–639. doi:10.1002/pbc.25862

25. Hettmer S, Li Z, Billin AN, Barr FG, Cornelison DDW, Ehrlich AR, et al. Rhabdomyosarcoma: Current Challenges and Their Implications for Developing Therapies. Cold Spring Harbor Perspectives in Medicine. 2014;4: a025650–a025650. doi:10.1101/cshperspect.a025650

26. Kikuchi K, Tsuchiya K, Otabe O, Gotoh T, Tamura S, Katsumi Y, et al. Effects of PAX3-FKHR on malignant phenotypes in alveolar rhabdomyosarcoma. Biochemical and Biophysical Research Communications. 2008;365: 568–574. doi:10.1016/j.bbrc.2007.11.017

27. Ginsberg JP, Davis RJ, Bennicelli JL, Ginsberg JP, Davis RJ, Bennicelli JL, et al. Up-Regulation of MET but not Neural Cell Adhesion Molecule Expression by the PAX3-FKHR Fusion Protein in Alveolar Rhabdomyosarcoma. Cancer Research. 1998;Aug 15: 3542–3546.

28. Strahm B, Durbin AD, Sexsmith E, Malkin D. The CXCR4-SDF1α axis is a critical mediator of rhabdomyosarcoma metastatic signaling induced by bone marrow stroma. Clinical & Experimental Metastasis. 2008;25: 1–10. doi:10.1007/s10585-007-9094-6

29. Jankowski K, Kucia M, Wysoczynski M, Reca R, Zhao D, Trzyna E, et al. Both Hepatocyte Growth Factor (HGF) and Stromal-Derived Factor-1 Regulate the Metastatic Behavior of Human Rhabdomyosarcoma Cells, but only HGF Enhances Their Resistance to Radiochemotherapy. Cancer Research. 2003;63: 7926–7935.

30. Thuault S, Hayashi S, Lagirand-Cantaloube J, Plutoni C, Comunale F, Delattre O, et al. P-cadherin is a direct PAX3–FOXO1A target involved in alveolar rhabdomyosarcoma aggressiveness. Oncogene. 2012; 1–12. doi:10.1038/onc.2012.217

Vanessa Ribes
Transcriptional network and cell fate IJM Group
IJM CNRS UMR7592, 15 rue Hélène Brion 75013 Paris
vanessa.ribes@ijm.fr
Tels: +33 (0) 1 57 27 81 94 ; +33 (0) 6 49 45 08 82

31. Abraham J, Nuñez-Álvarez Y, Hettmer S, Carrió E, Chen H-IHIH, Nishijo K, et al. Lineage of origin in rhabdomyosarcoma informs pharmacological response. Genes and Development. 2014;28: 1578–1591. doi:10.1101/gad.238733.114

32. Pandey PR, Chatterjee B, Olanich ME, Khan J, Miettinen MM, Hewitt SM, et al. PAX3-FOXO1 is essential for tumour initiation and maintenance but not recurrence in a human myoblast model of rhabdomyosarcoma. The Journal of Pathology. 2017;241: 626–637. doi:10.1002/path.4867

33. Naini S, Etheridge KT, Adam SJ, Qualman SJ, Bentley RC, Counter CM, et al. Defining the cooperative genetic changes that temporally drive alveolar rhabdomyosarcoma. Cancer Research. 2008;68: 9583–9588. doi:10.1158/0008-5472.CAN-07-6178

34. Kikuchi K, Taniguchi E, Chen H-IH, Svalina MN, Abraham J, Huang ET, et al. Rb1 loss modifies but does not initiate alveolar rhabdomyosarcoma. Skeletal muscle. 2013;3: 27. doi:10.1186/2044-5040-3-27

Referee Figure Legends

Referee Figure 1: SA-bGalactosidase activity and GFP detection on transverse sections of embryos at 48hpe with the indicated constructs. aer: apical ectodermal ridge.

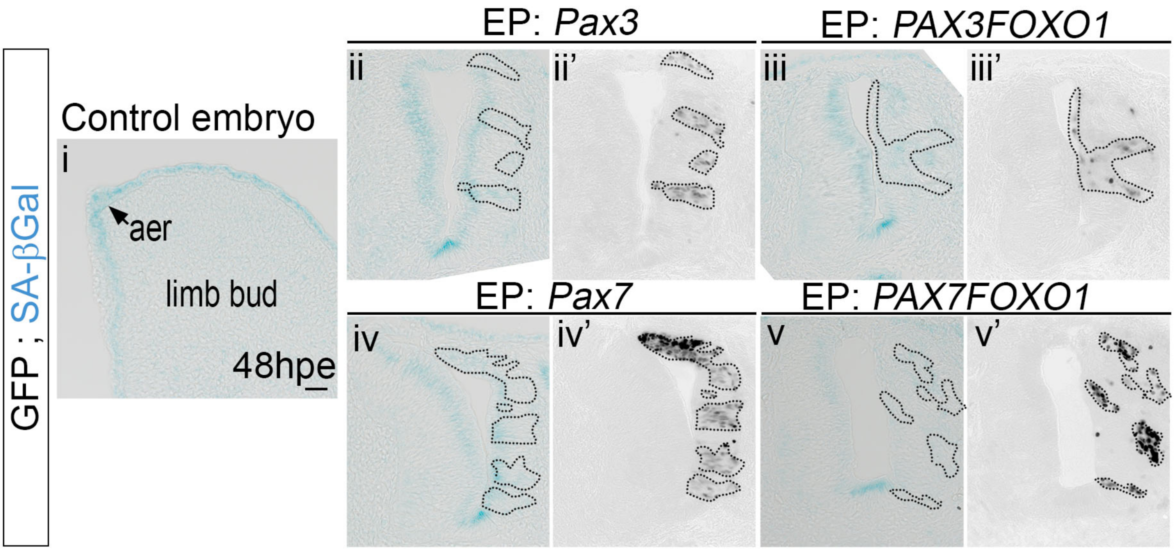

Curto et al., Referree Fig 1
