# Supplementary material for: The PAX-FOXO1s trigger fast trans-differentiation of chick embryonic neural cells into alveolar rhabdomyosarcoma with tissue invasive properties limited by S phase entry inhibition
Source: PLoS Genet. 2020 Nov 11;16(11):e1009164. doi: 10.1371/journal.pgen.1009164 (PMC7682867; doi:10.1371/journal.pgen.1009164)
Supplement: Attachment — Submitted filename: RMS_Curto_et_al_refereeletter_revision_round2-FINAL.pdf [file pgen.1009164.s020.pdf]

Vanessa Ribes
Transcriptional network and cell fate IJM Group
IJM CNRS UMR7592, 15 rue Hélène Brion 75013 Paris
vanessa.ribes@ijm.fr
Tels: +33 (0) 1 57 27 81 94 ; +33 (0) 6 49 45 08 82

We thank the referees and the editor for their careful examination of our manuscript and supportive comments. We have tackled all the issues raised. We have been straight to point. If required, we can provide a longer discussion to some of referee 2's comments. Changes incorporated are listed below and are highlighted in blue in the text.

Editor: Have all data underlying the figures and results presented in the manuscript been provided? Large-scale datasets should be made available via a public repository as described in the PLOS Genetics data availability policy, and numerical data that underlies graphs or summary statistics should be provided in spreadsheet form as supporting information.
All measures obtained on chick embryos and HFF cells can be found in S5 and S6 Tables.

Reviewer #1: In this revised version, Gonzalez Curto et al addressed all the concerns raised previously. Their rebuttal letter is very thorough, they took the time to provide a substantial point-by-point response addressing all the issues raised by the different reviewers.
They rearranged their figure panels and simplified the text. They opted to focus on PAX3-FOXO1, and they provided the different controls that were missing in the previous submission.
My only comment for the authors is that the Summit software is not designed and not appropriate to adequately measure cell cycle profiles. The use of a flow cytometry DNA analysis software like ModFit LT (or other) really makes a difference when it comes to cell cycle profile analyses. It is more accurate than simply separating your cells into three bins, as the software models the cells distribution into the different cell cycle phases. I recommend that the authors re-analyze their cell cycle data using such a software.
We have reanalysed our FCAS sorts using Flowjo™ to gate cell cycle phases based of cell DNA content, DNA was labelled using Vybrant dyecycle violet stain. We have used the Dean/Jett/Fox model to extrapolate the phases and adding a manual range on the G1 and G2 to compensate for variation in the position of the G1 peak in discrete lots of labelled cells. It is worth to note that in individual samples both control GFP$^-$ cells and *pCIG, Pax3* or *PAX3-FOXO1* expressing GFP$^+$ cells shared the same gating strategy.

Overall the results are in line with the one presented in our previous manuscript. In presence of PAX3-FOXO1 cells are more likely to be in the G1 phase than control pCIG or GFP$^-$ cells. Less cells are in the S and G2 phases. This phenotype is seen in presence of Pax3, yet is less severe.

In the new manuscript, the percentages of cells in each phase in Fig 6Cii-iv have been changed according to our new evaluation, so are the graphs in S6 Fig. In the latter, we have added panels presenting our gating strategy S6A Fig and the distribution of DNA content in both GFP$^+$ and GFP$^-$ cells (black line in S6B Fig). On these distributions the extrapolation curve obtained using the FlowJo model is represented in pink and the cell cycle distribution as well.

Reviewer #2: The revised manuscript has addressed a lot of the concerns bought up by the original review.
Several concerns remain.
The conclusion in the abstract "Together, our findings reveal a mechanism underpinning the apparent limited oncogenicity of PAXFOXO1 fusion transcription factors and support a neural origin for FP-RMS." **is too strong** given the lack of evidence provided by the authors of the neural origin, and the overwhelming evidence that the cell of origin is likely to be muscle progenitor cells for the majority of cases. The authors can say that their results show that "PAX3FOXO1 is sufficient to divert cells from a generic neurogenic program" not that it definitively proves a neural origin, given that the PAX3-FOXO1 fusion gene is a pioneering transcription factor able to divert almost any cell into a myogenic lineage, and also able to induce some neural genes given PAX3 is expressed in neural crest that the FOXO1 is a strong transactivator of all targets of PAX3.
We have rephrased the sentence in our abstract to match to the elements we have brought in our discussion and the issue raised by the referee: "Together, our findings reveal a mechanism that can

Vanessa Ribes
Transcriptional network and cell fate IJM Group
IJM CNRS UMR7592, 15 rue Hélène Brion 75013 Paris
vanessa.ribes@ijm.fr
Tels: +33 (0) 1 57 27 81 94 ; +33 (0) 6 49 45 08 82

explain the apparent limited oncogenicity of PAX-FOXO1 fusion transcription factors. They are also consistent with certain clinical reports indicative of a neural origin of FP-RMS."

We have discussed in our manuscript and in our previous letter to the referees the evidence from clinical and fundamental studies in line with the idea that FP-RMS could not only derive from muscle progenitors but also from other cell types, including mesenchymal stem cells, neural cells, including neural crest cells. To our knowledge, the similarities between the traits of FP-RMS and muscle progenitors (transcriptomic, bio-markers, DNA methylation state) have not yet allowed to distinguish whether FP-RMS and muscle progenitors share a common origin or whether FP-RMS cells have co-opted gene network controlling muscle cell identities. As mentioned by the referee and written in our discussion, the ability of PAX-FOXO1 to transform discrete cell types, taken together with their pioneer activity, is compatible with the second hypothesis. The location of tumours is arguing for a mesenchymal and muscle progenitor origin in most of the cases, but not all. Whether phenotypic inter-patient variation of FP-RMS could arise from discrete cell origin is still an open question. In the future, we hope to be able, with our chick model, to further assess whether the cell origin alter the response of cells to the fusion transcription factors and whether it will have incidence on the migration and tissue invasive properties of cells.

Other minor concerns:

1.Throughout the manuscript the authors should change PAX3FOXO1 to PAX3-FOXO1 etc., which is the more standard nomenclature.
This has been changed accordingly in the text and on figures.

2.The sentence "Genetically re-activating core cell cycle regulators lift up PAXFOXO1 mediated cell cycle inhibition" is awkward does the author mean rescue PAX-FOXO1 mediated cell cycle inhibition?
We have changed the sentence and have used as proposed the verb rescue.

3.The authors should cite PMID: 10557309 which was one of the first manuscripts to show the activation of a myogenic transcription program by PAX3-FOXO1.
In the introduction, the paper PMID: 10557309 is cited in the reviews we mentioned when referring on the role of PAX3-FOXO1 in the establishment of a FP-RMS like identity. In the discussion, we have introduced the paper PMID: 10557309, when we talk about co-option of development program mediated by PAX-FOXO1 activity.

4.The authors state "several of the target genes encode cell surface proteins required for FP-RMS cells migration". Change "required" to "associated with" because it is not clear that these genes are required for migration.
We have changed the sentence. "First, several of the target genes encode cell surface proteins which are key cell migration regulators and the alteration of the some of them was shown to affect RMS cell motility".

5.The authors state "FP-RMS cells harbour undifferentiated muscle cell determinants" not sure what this means, please expand.
To clarify, we reformulated: "FP-RMS cells express undifferentiated muscle cell master TFs".

6.The authors state " PAXFOXO1s perturb the core cell cycle machinery while PAX3FOXO1 activity during G2/M and G1/S checkpoints facilitates the amplification of transformed cells" I am unsure of the meaning of "facilitates the amplification of transformed cells".
To clarify, we have extended the description of the studies dealing with how PAX3-FOXO1 impact cell cycle progression.
We can read now: "Third, PAX-FOXO1s perturb the core cell cycle machinery [1,2]. Cross-interactions between PAX3-FOXO1 with the anti-apoptotic gene *BCL-XL* or the senescent factor $p16^{INK4A}$ promote

Vanessa Ribes
Transcriptional network and cell fate IJM Group
IJM CNRS UMR7592, 15 rue Hélène Brion 75013 Paris
vanessa.ribes@ijm.fr
Tels: +33 (0) 1 57 27 81 94 ; +33 (0) 6 49 45 08 82

cell survival [3–5]. PAX3-FOXO1 increases the proliferation of fibroblasts and myoblasts and this associated with a downregulation of cyclin-dependent kinase inhibitors (CDKN1B and CDKN1C) [6,7]. The fusion protein displays elevated levels in the G2 phase which are required for the upregulation of G2/M checkpoint adaptation genes [8]".

7. Figure 1 Legend The cervical to thoracic region (blue square) blue looks black not blue.
This indeed a "dark blue" square, we have changed the text accordingly.

8. In l Figure 1 Legend specify "hpe" means hours post electroporation.
We have added this specification to Figure 1 legend.

9. Several cell lines are mentioned which appear to be identical in Figure 2D cell e.g. lines RD and RDabl and RH30 and SJRh30. Please clarify why identical lines are used and add to manuscript.
The ERMS, RD and RDabl, and the ARMS, RH30 and SJRH30, are likely to be derived from the same patient ([9], ATCC STR profiles). Regardless their origin, RH30 and SJRH30 lines expressed discrete levels of PAX3-FOXO1 and were of interest to our study to look into at the correlation between those levels and that of the FP-RMS identity genes. The profiles of expression of all genes assessed in the RD and RDabl, and RH30 and SHRH30 show for many of them some significant variations (See for instance EYA2 profiles in Fig 2Di).

10. Generally, although the references to figures are better than the original manuscript there still is a poor referencing in the manuscript.
To tackle this issue, we have:

i) Stated that the non-electroporated side of the neural tube serve as an internal control to which the electroporated side can be compared to (see first paragraph in the result part).
ii) We have added new calls of individual figure panels in the result sections to remove any ambiguity. When necessary, we have indicated comparison between panels. For instance on page 12: one can read: "Paralleling PAX3-FOXO1 mediated cell fate changes, drastic rearrangement of the pseudo-stratified neuro-epithelium occurred (compare Fig 5Aiii,iii' to Fig 5Ai,i')."

11. The sentence "the histology of the transformed cells strongly support a routing of cells not towards a myoblast like state but a FP-RMS like state" on Page 38 Not sure what the authors mean as there is much evidence that PAX3-FOXO1 does route cells to a myoblastic like state.
We agree it was not clear. We wanted to compare to the shape of PAX3-FOXO1 expressing cells to the myoblasts derived from the somites, which are elongated.
We rephrased "In contrast, the presence of both myoblast and non myoblasts associated TFs within the chick neural cells expressing PAX3-FOXO1 and PAX7-FOXO1 support a routing of cells a FP-RMS like state."

12. The statement "This idea is further supported by the observation that the regulatory regions interacting with PAX3 promoter upon t(2;13)(q35;q14) translocations can remain active in the neural tube after the translocation" is ambiguous. What regions is the author is referring to? There is evidence that the FOXO1 region involved in the translocation is also active in myogenesis.
We have reformulated. 'This idea is further supported by the observation that the regulatory regions in the vicinity of the *PAX3* locus which are not impacted by the t(2;13)(q35;q14) translocations remain active in the neural tube after the translocation [10]'.

13. For the comment "These results provide insight for why complementation of PAXFOXO1s with genetic aberrations promoting cell cycle progression, such as the gain of MYCN or CCND1, or loss of p53 or RB1, can enhance their tumorigenic potential", please add CDKN2A loss.
This has been modified.

Vanessa Ribes
Transcriptional network and cell fate IJM Group
IJM CNRS UMR7592, 15 rue Hélène Brion 75013 Paris
vanessa.ribes@ijm.fr
Tels: +33 (0) 1 57 27 81 94 ; +33 (0) 6 49 45 08 82

Reviewer #3: I am pleased to note that the authors have satisfactorily addressed my comments, as well as most from the other reviewer. I am happy to now recommend this manuscript for publication in PLOS Genetics.

Thank you.

REFERENCES

1. Linardic CM. PAX3-FOXO1 fusion gene in rhabdomyosarcoma. Cancer letters. 2008;270: 10–8. doi:10.1016/j.canlet.2008.03.035
2. Marshall AD, Grosveld GC. Alveolar rhabdomyosarcoma - The molecular drivers of PAX3/7-FOXO1-induced tumorigenesis. Skeletal muscle. 2012;2: 25. doi:10.1186/2044-5040-2-25
3. Margue CM, Bernasconi M, Barr FG, Schafer BW. Transcriptional modulation of the anti-apoptotic protein BCL-XL by the paired box transcription factors PAX3 and PAX3/FKHR. Oncogene. 2000;19: 2921–2929. doi:10.1038/sj.onc.1203607
4. Ayyanathan K, Fredericks WJ, Berking C, Herlyn M, Balakrishnan C, Gunther E, et al. Hormone-dependent tumor regression in vivo by an inducible transcriptional repressor directed at the PAX3-FKHR oncogene. Cancer Research. 2000;60: 5803–5814.
5. Linardic CM, Naini S, Herndon JE, Kesserwan C, Qualman SJ, Counter CM. The PAX3-FKHR fusion gene of rhabdomyosarcoma cooperates with loss of p16INK4A to promote bypass of cellular senescence. Cancer Research. 2007;67: 6691–6699. doi:10.1158/0008-5472.CAN-06-3210
6. Zhang L, Wang C. PAX3-FKHR transformation increases 26 S proteasome-dependent degradation of p27Kip1, a potential role for elevated Skp2 expression. Journal of Biological Chemistry. 2003;278: 27–36. doi:10.1074/jbc.M205424200
7. Roeb W, Boyer A, Cavenee WK, Arden KC. PAX3-FOXO1 controls expression of the p57Kip2 cell-cycle regulator through degradation of EGR1. Proceedings of the National Academy of Sciences of the United States of America. 2007;104: 18085–90. doi:10.1073/pnas.0708910104
8. Kikuchi K, Hettmer S, Aslam MI, Michalek JE, Laub W, Wilky BA, et al. Cell-Cycle Dependent Expression of a Translocation- Mediated Fusion Oncogene Mediates Checkpoint Adaptation in Rhabdomyosarcoma. 2014;10. doi:10.1371/journal.pgen.1004107
9. Hinson ARP, Jones R, Crose LES, Belyea BC, Barr FG, Linardic CM. Human Rhabdomyosarcoma Cell Lines for Rhabdomyosarcoma Research: Utility and Pitfalls. Front Oncol. 2013;3. doi:10.3389/fonc.2013.00183
10. Vicente-García C, Villarejo-Balcells B, Irastorza-Azcárate I, Naranjo S, Acemel RD, Tena JJ, et al. Regulatory landscape fusion in rhabdomyosarcoma through interactions between the PAX3 promoter and FOXO1 regulatory elements. Genome Biology. 2017;18. doi:10.1186/s13059-017-1225-z
